# Learning Without Feedback: Fixed Random Learning Signals Allow for Feedforward Training of Deep Neural Networks

**DOI:** 10.3389/fnins.2021.629892

**Published:** 2021-02-10

**Authors:** Charlotte Frenkel, Martin Lefebvre, David Bol

**Affiliations:** ^1^Institute of Neuroinformatics, University of Zürich and ETH Zürich, Zurich, Switzerland; ^2^ICTEAM Institute, Université catholique de Louvain, Louvain-la-Neuve, Belgium

**Keywords:** backpropagation, deep neural networks, weight transport, update locking, edge computing, biologically-plausible learning

## Abstract

While the backpropagation of error algorithm enables deep neural network training, it implies (i) bidirectional synaptic weight transport and (ii) update locking until the forward and backward passes are completed. Not only do these constraints preclude biological plausibility, but they also hinder the development of low-cost adaptive smart sensors at the edge, as they severely constrain memory accesses and entail buffering overhead. In this work, we show that the one-hot-encoded labels provided in supervised classification problems, denoted as targets, can be viewed as a proxy for the error sign. Therefore, their fixed random projections enable a layerwise feedforward training of the hidden layers, thus solving the weight transport and update locking problems while relaxing the computational and memory requirements. Based on these observations, we propose the direct random target projection (DRTP) algorithm and demonstrate that it provides a tradeoff between accuracy and computational cost that is suitable for adaptive edge computing devices.

## 1. Introduction

Artificial neural networks (ANNs) were proposed as a first step toward bio-inspired computation by emulating the way the brain processes information with densely-interconnected neurons and synapses as computational and memory elements, respectively (Rosenblatt, 1962; Bassett and Bullmore, 2006). In order to train ANNs, it is necessary to identify how much each neuron contributed to the output error, a problem referred to as the *credit assignment* (Minsky, 1961). The backpropagation of error (BP) algorithm (Rumelhart et al., 1986) allowed solving the credit assignment problem for multi-layer ANNs, thus enabling the development of deep networks for applications ranging from computer vision (Krizhevsky et al., 2012; LeCun et al., 2015; He et al., 2016) to natural language processing (Hinton et al., 2012; Amodei et al., 2016). However, two critical issues preclude BP from being biologically plausible.

**Figure 1:**
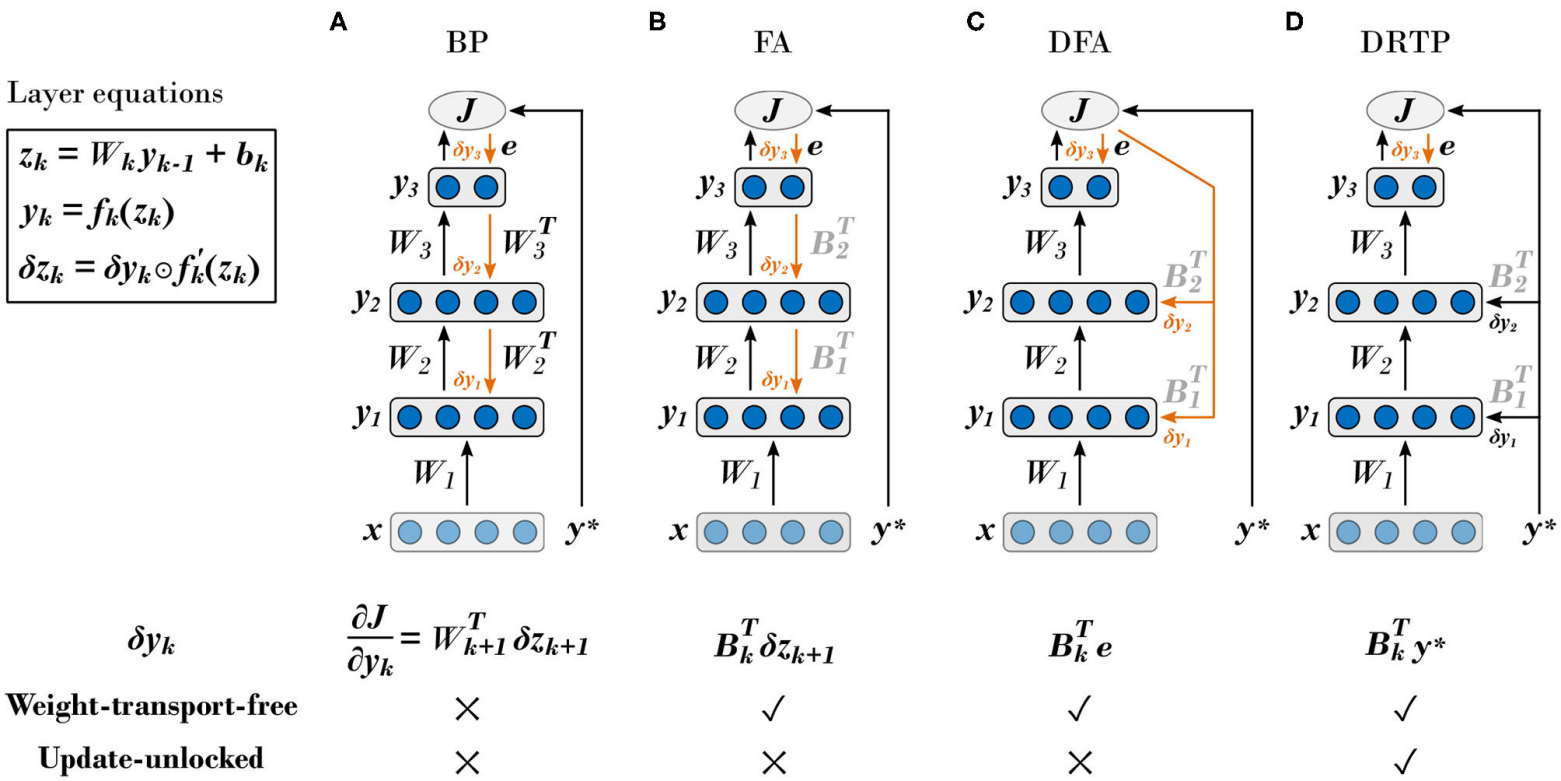
The proposed direct random target projection algorithm builds on feedback-alignment-based algorithms to tackle the weight transport problem while further releasing update locking. Black arrows indicate the feedforward pathways and orange arrows the feedback pathways. In the *k*-th layer, the weighted sum of inputs *y*_*k*−1_ is denoted as *z*_*k*_ , the bias as *b*_*k*_ , the activation function as *f*_*k*_(·) and its derivative as $f_{k}^{\prime }\left(\cdot \right)$, with *k* ∈ [1, *K*], *k* ∈ ℕ, and *K* the number of layers. Trainable forward weight matrices are denoted as *W*_*k*_ and fixed random connectivity matrices as *B*_*k*_ . The input vector is denoted as *x*, the target vector as y^*^ and the loss function as *J*(·). The estimated loss gradients for the outputs of the *k*-th hidden layer, denoted as δ*y*_*k*_, are provided for each training algorithm. The layer equations for *z*_*k*_ , *y*_*k*_ and δ*z*_*k*_ , defined as the modulatory signals, are provided in the upper left corner, with ⊙ denoting the elementwise multiplication operator. **(A)** Backpropagation of error (BP) algorithm (Rumelhart et al., 1986). **(B)** Feedback alignment (FA) algorithm (Lillicrap et al., 2016). **(C)** Direct feedback alignment (DFA) algorithm (Nøkland, 2016). **(D)** Proposed direct random target projection (DRTP) algorithm. Adapted from Nøkland (2016) and Czarnecki et al. (2017).

First, BP requires symmetry between the forward and backward weights, which is known as the *weight transport problem* (Grossberg, 1987). Beyond implying a perfect and instantaneous communication of parameters between the feedforward and feedback pathways, error backpropagation requires each layer to have full knowledge of all the weights in the downstream layers, making BP a non-local algorithm for both weight and error information. From a hardware efficiency point of view, the weight symmetry requirement also severely constrains memory access patterns (Crafton et al., 2019). Therefore, there is an increasing interest in developing training algorithms that release this constraint, as it has been shown that weight symmetry is not mandatory to reach near-BP performance (Liao et al., 2016). The feedback alignment (FA) algorithm (Lillicrap et al., 2016), also called random backpropagation (Baldi et al., 2018), demonstrates that using fixed random weights in the feedback pathway allows conveying useful error gradient information: the network learns to align the forward weights with the backward ones. Direct feedback alignment (DFA) (Nøkland, 2016) builds on these results and directly propagates the error between the network predictions and the *targets* (i.e. one-hot-encoded labels) to each hidden layer through fixed random connectivity matrices. DFA demonstrates a limited accuracy penalty compared to BP on the MNIST (LeCun et al., 1998) and CIFAR-10 (Krizhevsky et al., 2009) datasets, while using the output error as a global modulator and keeping weight information local. Therefore, DFA bears important structural similarity with learning rules that are believed to take place in the brain (Guerguiev et al., 2017; Neftci et al., 2017), known as three-factor synaptic plasticity rules, which rely on local pre- and post-synaptic spike-based activity together with a global modulation (Urbanczik and Senn, 2014). Finally, another approach for solving the weight transport problem consists in computing targets for each layer instead of gradients. The target values can either be computed based on auto-encoders at each layer (Lee et al., 2015) or generated by making use of the pre-activation of the current layer and the error of the next layer, propagated through a dedicated trainable feedback pathway (Ororbia and Mali, 2019). The BP, FA and DFA algorithms are summarized in Figures 1A–C, respectively.

The second issue of BP is its requirement for a full forward pass before parameters can be updated during the backward pass, a phenomenon referred to as *update locking* (Czarnecki et al., 2017; Jaderberg et al., 2017). Beyond making BP biologically implausible, update locking has critical implications for BP implementation as it requires buffering all the layer inputs and activations during the forward and backward passes in order to compute the weight updates, leading to a high memory overhead. As the previously-described FA and DFA solutions to the weight transport problem only tackle the weight locality aspect, specific techniques enabling local error handling or gradient approximation are required to tackle update locking. On the one hand, the *error locality approach* relies on layerwise loss functions (Mostafa et al., 2018; Belilovsky et al., 2019; Nøkland and Eidnes, 2019; Kaiser et al., 2020), it enables training layers independently and without requiring a forward pass in the entire network. The generation of local errors can be achieved with auxiliary fixed random classifiers, allowing for near-BP performance on the MNIST and CIFAR-10 datasets (Mostafa et al., 2018). This strategy has also been ported to a biologically-plausible spike-based three-factor synaptic plasticity rule (Kaiser et al., 2020). Scaling to ImageNet (Deng et al., 2009) requires either the use of two combined layerwise loss functions (Nøkland and Eidnes, 2019) or a parallel optimization of a greedy objective using deeper auxiliary classifiers (Belilovsky et al., 2019). However, the error locality approach still suffers from update locking at the layer scale as layerwise forward and backward passes are required. Beyond implying a computational overhead, the auxiliary classifiers also suffer from the weight transport problem, a requirement that can only be partially relaxed: in order to maintain performance, it is necessary to keep at least the weight sign information during the layerwise backward passes (Mostafa et al., 2018). On the other hand, the *synthetic gradients approach* (Czarnecki et al., 2017; Jaderberg et al., 2017) relies on layerwise predictors of subsequent network computation. However, training local gradient predictors still requires backpropagating gradient information from deeper layers.

In order to fully solve both the weight transport and the update locking problems, we propose the direct random target projection (DRTP) algorithm (Figure 1D). Compared to DFA, the targets are used in place of the output error and projected onto the hidden layers. We demonstrate both theoretically and experimentally that, in the framework of classification problems, the error sign information contained in the targets is sufficient to maintain feedback alignment with the loss gradients δ*z*_*k*_ for the weighted sum of inputs in layer *k*, denoted as the *modulatory signals* in the subsequent text, and allows training multi-layer networks, leading to three key advantages. First, DRTP solves the weight transport problem by entirely removing the need for dedicated feedback pathways. Second, layers can be updated independently and without update locking as a full forward pass is not required, thus reducing memory requirements by releasing the need to buffer inputs and activations of each layer. Third, DRTP is a purely feedforward and low-cost algorithm whose updates rely on layerwise information that is immediately available upon computation of the layer outputs. Estimating the layerwise loss gradients δ*y*_*k*_ only requires a label-dependent random vector selection, contrasting with the error locality and synthetic gradients approaches that require the addition of side networks for error or gradient prediction. DRTP even compares favorably to DFA, as the latter still requires a multiplication between the output error and a fixed random matrix.

Therefore, DRTP allows relaxing structural, memory and computational requirements, yet we demonstrate that, compared to BP, FA and DFA, DRTP is ideal for implementation in edge-computing devices, thus enabling adaptation to uncontrolled environments while meeting stringent power and resource constraints. Suitable applications for DRTP range from distributed smart sensor networks for the Internet-of-Things (IoT) (Bol et al., 2015) to embedded systems and cognitive robotic agents (Milde et al., 2017). The MNIST and CIFAR-10 datasets have thus been selected for benchmarking as they are representative of the complexity level required in autonomous always-on adaptive edge computing, which is not the case of larger and more challenging datasets such as ImageNet. This furthermore highlights that edge computing is an ideal use case for biologically-motivated algorithms, as an out-of-the-box application of feedback-alignment- and target-propagation-based algorithms currently does not scale to complex datasets (see Bartunov et al., 2018 for a recent review). We demonstrate this claim in Frenkel et al. (2020) with the design of an event-driven convolutional processor that requires only 16.8–% power and 11.8–% silicon area overheads for on-chip online learning, a record-low overhead that is specifically enabled by DRTP, thus highlighting its low cost for edge computing devices. Finally, as DRTP can also be formulated as a three-factor learning rule for biologically-plausible learning, it is suitable for embedded neuromorphic computing, in which high-density synaptic plasticity can currently not be achieved without compromising learning performance (Frenkel et al., 2019b,c).

## 2. Results

### 2.1. Weight Updates Based Only on the Error Sign Provide Learning to Multi-Layer Networks

We demonstrate with two experiments, respectively on a regression task and a classification problem, that modulatory signals based only on the error sign are within 90° of those prescribed by BP, thus providing learning in multi-layer networks. To do so, we use an error-sign-based version of DFA, subsequently denoted as sDFA, in which the error vector is replaced by the error sign in the global feedback pathway.

#### 2.1.1. Regression

This first experiment aims at demonstrating that the error sign provides useful modulatory signals to multi-layer networks by comparing training algorithms on a regression task. The objective is to approximate 10 non-linear functions $T_{j}\left(x\right)=\text{cos}\left(\bar{x}+\phi_{j}\right)$, where ϕ_*j*_ = −π/2+*jπ*/9 for *j* ∈ [0, 9], *j* ∈ ℕ_0_ and $\bar{x}$ denotes the mean of *x*, a 256-dimensional vector whose entries are drawn from a normal distribution with a mean lying in [−π, π] (see section 4). A 256-100-100-10 fully-connected network is trained to approximate *T*(·) with five training algorithms: shallow learning (i.e. frozen random hidden layers and a trained output layer), BP, FA, DFA and sDFA.

**Figure 2:**
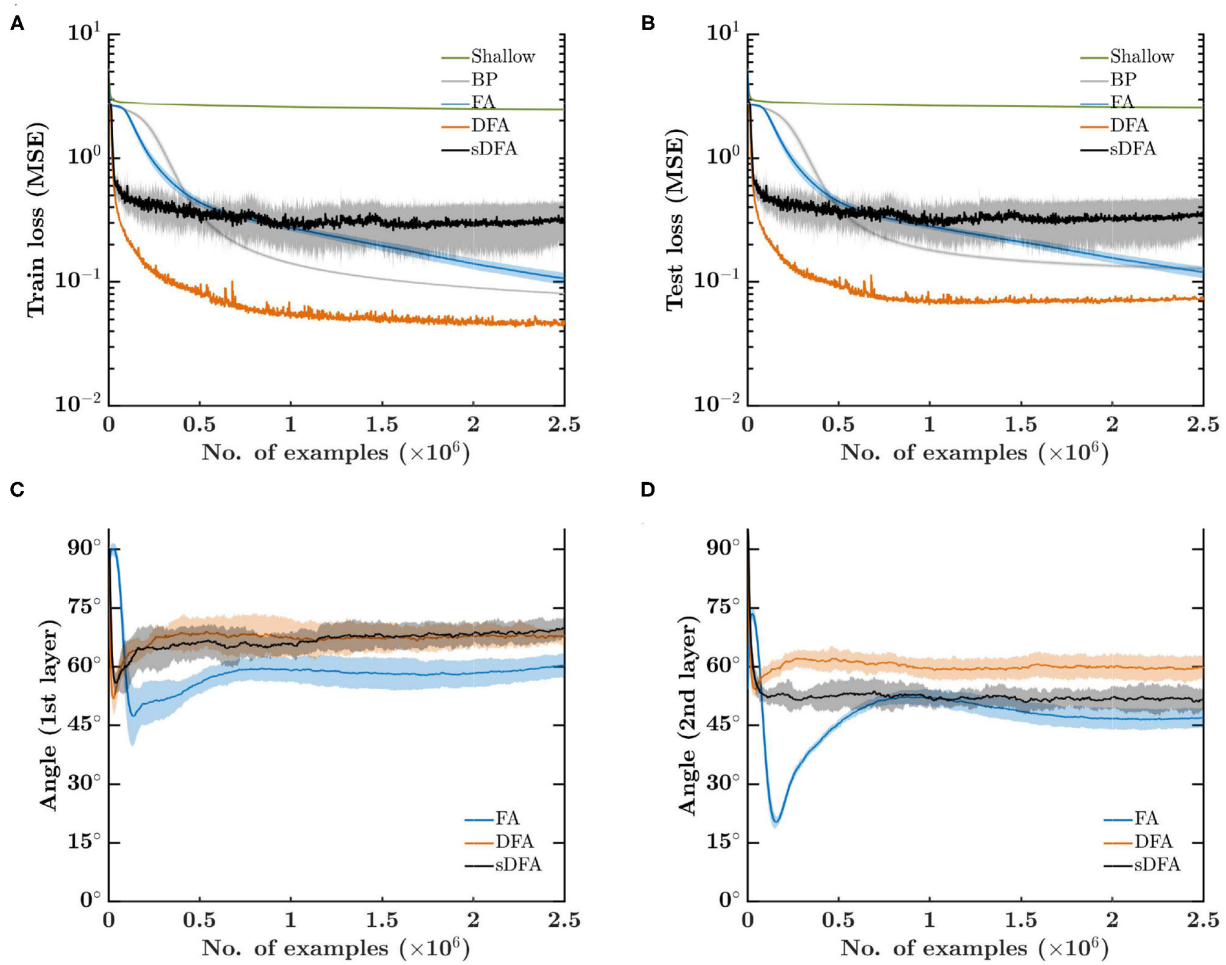
Error-sign-based direct feedback alignment (sDFA) provides useful modulatory signals in regression tasks. A 256-100-100-10 network with tanh hidden and output units is trained to learn cosine functions with five training algorithms: shallow learning, BP, FA, DFA and sDFA. With this simple setup, BP and FA suffer from the vanishing gradients problem, which would be alleviated by using ReLU-based networks with batch normalization. The scope of the figure is to highlight that sDFA provides useful modulatory signals for regression tasks, without any additional technique. As for other feedback-alignment-based algorithms, sDFA updates are within 90° of the backpropagation updates. The train and test losses and the alignment angles are monitored every 1k samples, error bars are one standard deviation over 10 runs. Angles have been smoothed by an exponentially-weighted moving average filter with a momentum coefficient of 0.95. **(A)** Mean squared error loss on the 5k-example training set. **(B)** Mean squared error loss on the 1k-example test set. **(C)** Angle between the modulatory signals δ*z*_*k*_ prescribed by BP and by feedback-alignment-based algorithms in the first hidden layer. **(D)** Angle between the modulatory signals δ*z*_*k*_ prescribed by BP and by feedback-alignment-based algorithms in the second hidden layer.

The mean squared error (MSE) loss on the training set is shown in Figure 2A. While shallow learning fails to learn a meaningful approximation of *T*(·), sDFA and DFA show the fastest initial convergence due to the separate direct feedback pathway precluding gradients from vanishing, which is clearly an issue for BP and FA. Although this would be alleviated by using ReLU-based networks with batch normalization (Ioffe and Szegedy, 2015), it highlights that direct-feedback-alignment-based methods do not need further techniques such as batch normalization to address this issue, ultimately leading to reduced hardware requirements. While DFA demonstrates the highest performance on this task, sDFA comes earlier to stagnation as it does not account for the output error magnitude reduction as training progresses, thus preventing a reduction of the effective learning rate in the hidden layers as the output error decreases. sDFA could therefore benefit from the use of a learning rate scheduler. Similar conclusions hold for the loss on the test set (Figure 2B). The angle between the modulatory signals prescribed by BP and by feedback-alignment-based algorithms is shown in Figures 2C,D for the first and second hidden layers, respectively. While all feedback-alignment-based algorithms lie close to each other within 90° of the BP modulatory signals, FA has a clear advantage during the first 100 epochs on the 5k-example training set. sDFA performs on par with DFA in the first hidden layer, while it surprisingly provides a better alignment than DFA in the second hidden layer, though not fully leveraged due to the absence of modulation in the magnitude of the updates from the output error.

#### 2.1.2. Classification

With this second experiment, we demonstrate that, in addition to providing useful modulatory signals for regression problems, the error sign information allows training multi-layer networks to solve classification problems. The task consists in training a 256-500-500-10 network to solve a synthetic classification problem with 16×16-pixel images and 10 classes; the data to classify is generated automatically with the Python sklearn library (Pedregosa et al., 2011) (see section 4). As for regression, the network is trained with shallow learning, BP, FA, DFA and sDFA.

**Figure 3:**
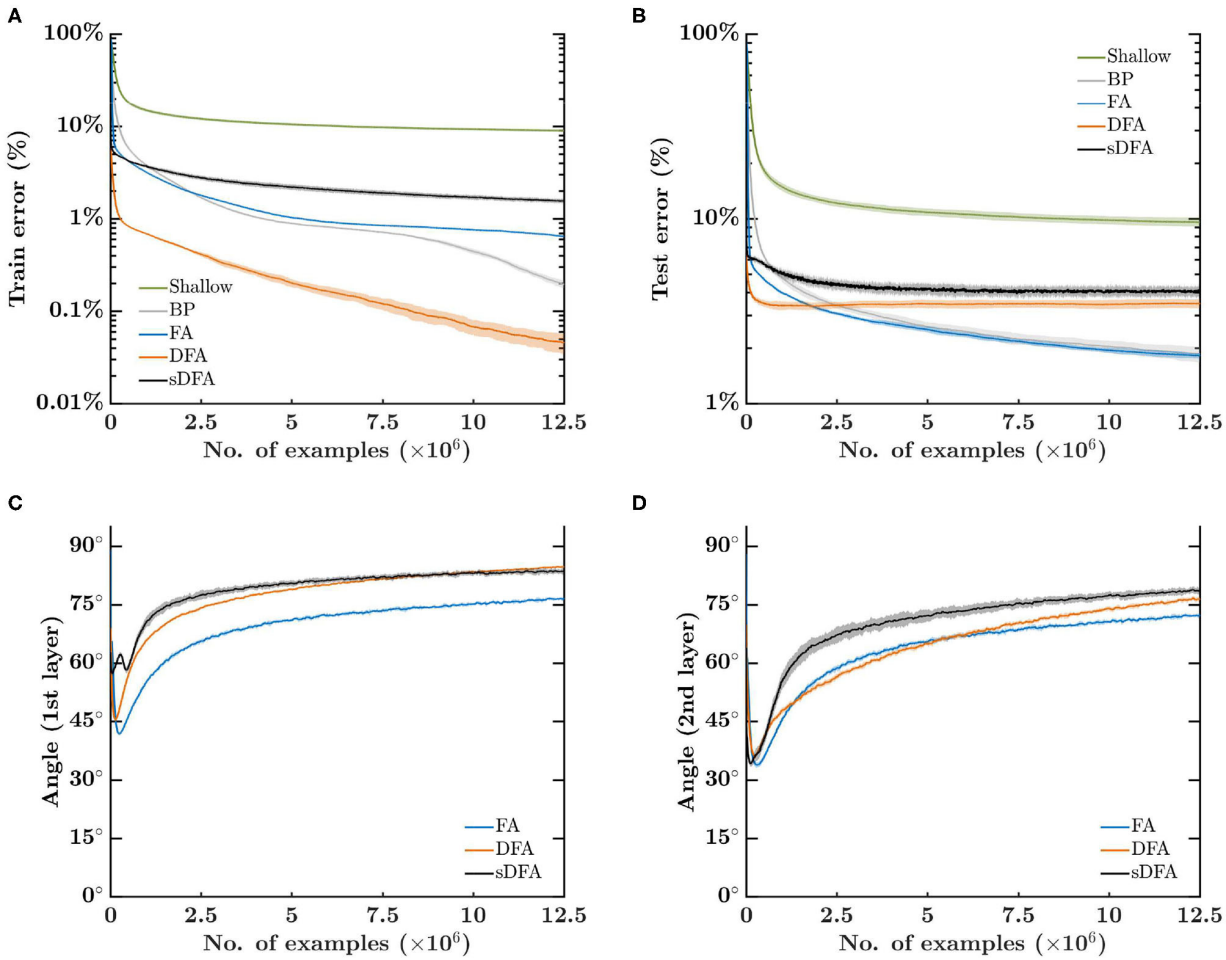
Error-sign-based direct feedback alignment (sDFA) provides useful modulatory signals in classification tasks. A 256-500-500-10 network with tanh hidden units and sigmoid output units is trained to classify a synthetic dataset of 16×16-pixel images into 10 classes with five training algorithms: shallow learning, BP, FA, DFA and sDFA. With this simple setup, BP and FA suffer from the vanishing gradients problem, which would be alleviated by using ReLU-based networks with batch normalization. The scope of the figure is to highlight that sDFA provides useful modulatory signals for classification tasks, without any additional technique. The update directions of the sDFA algorithm are within 90° of the backpropagation updates and are comparable to other feedback-alignment-based algorithms. The train and test losses and the alignment angles are monitored every 2.5k samples, error bars are one standard deviation over 10 runs. Angles have been smoothed by an exponentially-weighted moving average filter with a momentum coefficient of 0.95. **(A)** Error on the 25k-example training set, reaching on average 0.19% for BP, 0.64% for FA, 0.05% for DFA, 1.54% for sDFA and 8.95% for shallow learning after 500 epochs. **(B)** Error on the test set, reaching on average 1.85% for BP, 1.81% for FA, 3.48% for DFA, 4.07% for sDFA and 9.57% for shallow learning after 500 epochs. **(C)** Angle between the modulatory signals δ*z*_*k*_ prescribed by BP and by feedback-alignment-based algorithms in the first hidden layer. **(D)** Angle between the modulatory signals δ*z*_*k*_ prescribed by BP and by feedback-alignment-based algorithms in the second hidden layer.

Figure 3A shows that, after 500 epochs with a 25k-example training set, DFA provides the fastest and most accurate training with a classification error of 0.05%, followed by BP, FA and sDFA with 0.19, 0.64, and 1.54%, respectively. Shallow learning lags almost an order of magnitude behind with 8.95%. However, Figure 3B shows that DFA also has a higher overfitting and lies close to sDFA on the test set, with 3.48 and 4.07%, respectively. The lowest classification errors are of 1.85% for BP and 1.81% for FA, while shallow learning lags behind at 9.57%. The angle between the modulatory signals prescribed by BP and by feedback-alignment-based algorithms is shown in Figures 3C,D, for the first and second hidden layers, respectively. As for the regression task, all feedback-alignment-based algorithms exhibit alignments close to each other, while the convergence of BP and FA is slowed down by the vanishing gradients problem. Here, alignments tend to level off after 50 epochs, with the lowest angle provided by FA, followed by DFA and sDFA. As sDFA is always within 90° of the BP modulatory signals, it is able to train multi-layer networks.

### 2.2. For Classification Problems, a Feedback Pathway Is No Longer Required as the Error Sign Is Known in Advance

In the framework of classification problems, training examples (*x*, c^*^) consist of an input data sample to classify, denoted as *x*, and a label c^*^ denoting the class *x* belongs to, among *C* possible classes. The target vector, denoted as y^*^, corresponds to the one-hot-encoded class label c^*^. The output layer non-linearity must be chosen as a sigmoid or a softmax function, yielding output values that are strictly bounded between 0 and 1. Denoting the output vector of a *K*-layer network as *y*_*K*_, the error vector is defined as $e=y^{*}-y_{K}$. Under the aforementioned conditions, it results that the *c*-th entry of the *C*-dimensional error vector *e*, denoted *e*_*c*_, is defined as

$$\begin{array}{l} e_{c}=\{\begin{array}{cc} 1-y_{Kc} & \text{if }c=c^{*},\\ -y_{Kc} & \text{otherwise}. \end{array} \end{array}$$

As the entries of *y*_*K*_ are strictly bounded between 0 and 1, the error sign is given by

$$\begin{array}{l} e_{c}=\{\begin{array}{rr} 1 & \text{if }c=c^{*},\\ -1 & \text{otherwise}. \end{array} \end{array}$$

Due to the non-linearity in the output layer forcing the output values to remain strictly bounded between 0 and 1, the error sign is class-dependent and known in advance as training examples (*x*, c^*^) already provide the error sign information with the label c^*^. A feedback pathway is thus no longer required as we have shown that the error sign allows providing useful modulatory signals to train multi-layer networks. Therefore, beyond being free from the *weight transport problem* as DFA, sDFA also allows releasing *update locking* and the associated memory overhead in classification problems.

### 2.3. Direct Random Target Projection Delivers Useful Modulatory Signals for Classification

This section provides the grounds to show why the proposed direct random target projection (DRTP) algorithm delivers useful modulatory signals to multi-layer networks in the framework of classification problems. First, we show how DRTP can be viewed as a simplified version of sDFA in which the target vector y^*^ is used as a surrogate for the error sign. Next, we demonstrate mathematically that, in a multi-layer network composed of linear hidden layers and a non-linear output layer, the modulatory signals prescribed by DRTP and BP are always within 90° of each other, thus providing learning in multi-layer networks.

#### 2.3.1. DRTP Is a Simplified Version of Error-Sign-Based DFA

As we have shown that sDFA solves both the *weight transport* and the *update locking* problems in classification tasks, we propose the direct random target projection (DRTP) algorithm, illustrated in Figure 1D and written in pseudocode in Algorithm 1, as a simplified version of sDFA that enhances both performance and computational efficiency. In sDFA, the feedback signal randomly projected to the hidden layers is the sign of the error vector $e=y^{*}-y_{K}$, while in DRTP, this feedback signal is replaced by the target vector y^*^. Being a one-hot encoding of c^*^, y^*^ has a single positive entry corresponding to the correct class and zero entries elsewhere:

$$\begin{array}{l} y_{c}^{*}=\frac{1+\text{sign}(e_{c})}{2}=\{\begin{array}{cc} 1 & \text{if }c=c^{*},\\ 0 & \text{otherwise}. \end{array} \end{array}$$

**Table:** **Algorithm 1:** Pseudocode for the direct random target projection (DRTP) algorithm. *k* ∈ [1, *K*], *k* ∈ ℕ, denotes the layer index and *W*_*k*_, *b*_*k*_, *B*_*k*_ and *f*_*k*_(·) denote the trainable forward weights and biases, the fixed random connectivity matrices and the activation function of the *k*-th hidden layer, respectively. The weighted sum of inputs or pre-activation is denoted as *z*_*k*_ and the layer output or post-activation is denoted as *y*_*k*_, with *y*_0_ corresponding to the input *x*. The one-hot-encoding of labels among *C* output classes is denoted as y^*^ and the learning rate as η. The update for the weights and biases in the output layer are computed for sigmoid/softmax output units with a binary/categorical cross-entropy loss.

**for** (*k* = 1;*k* ≤ *K*; *k* = *k*+1) **do**
*z*_*k*_ ← *W*_*k*_*y*_*k*−1_ + *b*_*k*_
*y*_*k*_ ← *f*_*k*_(*z*_*k*_)
if *k* < *K* **then**
$W_{k}\leftarrow W_{k}+\eta \left(B_{k}^{T}y^{*}⊙f_{k}^{\prime }\left(z_{k}\right)\right)y_{k-1}^{T}$
$b_{k}\leftarrow b_{k}+\eta \left(B_{k}^{T}y^{*}⊙f_{k}^{\prime }\left(z_{k}\right)\right)$
**else**
$W_{K}\leftarrow W_{K}+\frac{\eta }{C}\left(y^{*}-y_{K}\right)y_{k-1}^{T}$
$b_{K}\leftarrow b_{K}+\frac{\eta }{C}\left(y^{*}-y_{K}\right)$
**end if**
**end for**

Thus, y^*^ corresponds to a surrogate for the error sign vector used in sDFA, where shift and rescaling operations have been applied to sign(*e*). As the connectivity matrices *B*_*k*_ in the DRTP gradients $\delta y_{k}=B_{k}^{T}y^{*}$ are fixed and random (Figure 1D), they can be viewed as comprising the rescaling operation. Only the shift operation applied to sign(*e*) makes a critical difference between DRTP and sDFA, which is favorable to DRTP for two reasons. First, DRTP is computationally cheaper than sDFA. Indeed, projecting the target vector y^*^ to the hidden layers through fixed random connectivity matrices is equivalent to a label-dependent selection of a layerwise random vector. On the contrary, sDFA requires multiplying the error sign vector with the fixed random connectivity matrices for each training example, as all entries of the error sign vector are non-zero. Second, experiments on the MNIST and CIFAR-10 datasets show that DRTP systematically outperforms sDFA (Supplementary Figures 1A, 2A, Supplementary Tables 1, 2). Indeed, when the feedback information only relies on the error sign and no longer on its magnitude, the weight updates become less selective to the useful information: as all entries of the error sign vector have unit norm, the *C*−1 entries corresponding to incorrect classes outweigh the single entry associated to the correct class and degrade the alignment (Supplementary Figures 1B, 2B).

#### 2.3.2. The Directions of the DRTP and BP Modulatory Signals Are Within 90° of Each Other

We provide a mathematical proof of alignment between the DRTP and BP modulatory signals. The structure of our proof is inspired from the FA proof of alignment in Lillicrap et al. (2016), which we expand in two ways. First, we extend this proof for the case of DRTP. Second, while Lillicrap et al. (2016) demonstrate the alignment with the BP modulatory signals for a network consisting of a single linear hidden layer, a linear output layer and a mean squared error loss, we demonstrate that alignment can be achieved for an arbitrary number of linear hidden layers, a non-linear output layer with sigmoid/softmax activation and a binary/categorical cross-entropy loss for classification problems. Both proofs are restricted to the case of a single training example. Under these conditions, it is possible to guarantee that the DRTP modulatory signals are aligned with those of BP. This comes from the fact that the prescribed weight updates lead to a soft alignment between the product of forward weight matrices and the fixed random connectivity matrices. The mathematical details, including the lemma and theorem proofs, have been abstracted out to the Supplementary Note 1.

**Figure 4:**
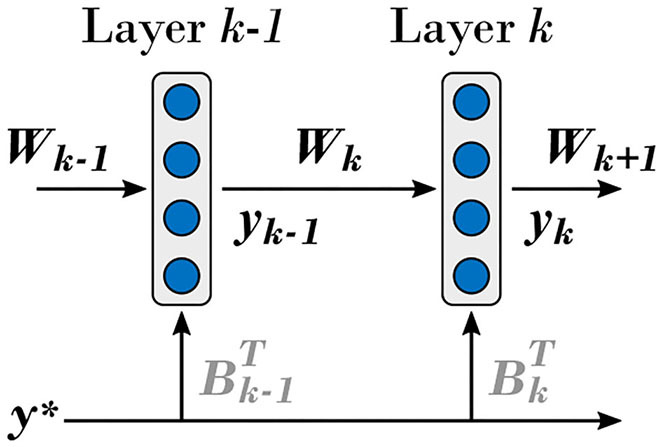
Network of DRTP-updated linear hidden layers considered in the context of the mathematical proof of alignment between the DRTP and BP modulatory signals. The same conventions as in Figure 1 are used.

In the case of the multi-layer neural network composed of linear hidden layers shown in Figure 4, the output of the *k*-th hidden layer is given by

$$\begin{array}{l} y_{k}=z_{k}=W_{k}y_{k-1}\;\text{for }k\in \left[1,K-1\right], \end{array}$$

where *K* is the number of layers, *y*_0_ = *x* is the input vector, and the bias vector *b*_*k*_ is omitted without loss of generality. The output layer is described by

$$\begin{array}{ll} z_{K} & =W_{K}y_{K-1},\\ y_{K} & =\sigma \left(z_{K}\right), \end{array}$$

where σ(·) is either the sigmoid or the softmax activation function. The loss function *J*(·) is either the binary cross-entropy (BCE) loss for sigmoid output units or the categorical cross-entropy (CCE) loss for softmax output units, computed over the *C* output classes:

$$\begin{array}{l} J_{\text{BCE}}\left(y_{K},y^{*}\right)=-\frac{1}{C}\overset{C}{\underset{c=1}{\sum }}(y_{c}^{*}\log\left(y_{Kc}\right)+\left(1-y_{c}^{*}\right)\log\left(1-y_{Kc}\right)),\\ J_{\text{CCE}}\left(y_{K},y^{*}\right)=-\frac{1}{C}\overset{C}{\underset{c=1}{\sum }}(y_{c}^{*}\log\left(y_{Kc}\right)). \end{array}$$

**Lemma**. In the case of zero-initialized weights, i.e. $W_{k}^{0}=0$ for *k* ∈ [1, *K*], *k* ∈ ℕ, and hence of zero-initialized hidden layer outputs, i.e. $y_{k}^{0}=0$ for *k* ∈ [1, *K*−1] and $z_{K}^{0}=0$, considering a DRTP-based training performed recursively with a single element of the training set (x, c^*^) and y^*^ denoting the one-hot encoding of c^*^, at every discrete update step *t*, there are non-negative scalars $s_{y_{k}}^{t}$ and $s_{W_{k}}^{t}$ for *k* ∈ [1, *K*−1] and a *C*-dimensional vector $s_{W_{K}}^{t}$ such that

$$\begin{array}{l} y_{k}^{t}=-s_{y_{k}}^{t}\left(B_{k}^{T}y^{*}\right)\;\text{for }k\in \left[1,K-1\right]\\ W_{1}^{t}=-s_{W_{1}}^{t}\left(B_{1}^{T}y^{*}\right)x^{T}\\ W_{k}^{t}=s_{W_{k}}^{t}\left(B_{k}^{T}y^{*}\right){\left(B_{k-1}^{T}y^{*}\right)}^{T}\;\text{for }k\in \left[2,K-1\right]\\ W_{K}^{t}=-s_{W_{K}}^{t}{\left(B_{K-1}^{T}y^{*}\right)}^{T}. \end{array}$$

**Theorem**. Under the same conditions as in the lemma and for the linear-hidden-layer network dynamics described above, the *k*-th layer modulatory signals prescribed by DRTP are always a negative scalar multiple of the Moore-Penrose pseudo-inverse of the product of forward matrices of layers *k*+1 to *K*, located in the feedback pathway between the output layer and the *k*-th hidden layer, multiplied by the error. That is, for *k* ∈ [1, *K*−1] and *t* > 0,

$$\begin{array}{l} -\frac{1}{s_{k}^{t}}{\left(\overset{k+1}{\underset{i=K}{\prod }}W_{i}^{t}\right)}^{+}e=B_{k}^{T}y^{*}\;\text{with }s_{k}^{t}>0. \end{array}$$

**Alignment**. In the framework of classification problems, as the coefficients $s_{k}^{t}$ are strictly positive scalars for *t* > 0, it results from the theorem that the dot product between the BP and DRTP modulatory signals is strictly positive, i.e.

$$\begin{array}{l} \;-e^{T}{\left(\overset{K}{\underset{i=k+1}{\prod }}W_{i}^{T}\right)}^{T}\left(B_{k}^{T}y^{*}\right)>0\\ e^{T}\underset{I}{\underset{︸}{{\left(\overset{K}{\underset{i=k+1}{\prod }}W_{i}^{T}\right)}^{T}{\left(\overset{k+1}{\underset{i=K}{\prod }}W_{i}\right)}^{+}}}\frac{e}{s_{k}^{t}}>0\\ \;\frac{e^{T}e}{s_{k}^{t}}>0. \end{array}$$

The BP and DRTP modulatory signals are thus within 90° of each other. □

### 2.4. DRTP Learns to Classify MNIST and CIFAR-10 Images Without Feedback

In this section, we compare DRTP with BP and other feedback-alignment-based algorithms, namely FA and DFA, on the MNIST and CIFAR-10 datasets. Both datasets have 10 output classes, they respectively consist in classifying 28×28 grayscale images of handwritten digits for MNIST and 32×32 RGB images of vehicles and animals for CIFAR-10. The network topologies considered in our experiments are, on the one hand, fully-connected (FC) networks with one or two hidden layers, respectively denoted as FC1 and FC2, each hidden layer being constituted of either 500 or 1,000 tanh units. On the other hand, convolutional (CONV) networks are used with either fixed random or trainable kernels. The CONV network for MNIST consists of one convolutional layer followed by a max-pooling layer and one fully-connected hidden layer, while for CIFAR-10 it consists of two convolutional layers, each followed by a max-pooling layer, and two fully-connected hidden layers (see section 4).

#### 2.4.1. MNIST

The results on the MNIST dataset are summarized in Table 1. In FC networks, BP, FA and DFA perform similarly, the accuracy degradation of FA and DFA is marginal. While there is a higher accuracy degradation for DRTP, it compares favorably to shallow learning, which suffers from a high accuracy penalty. It shows that DRTP allows training hidden layers to learn MNIST digit classification without feedback. The CONV network topology leads to the lowest error, highlighting that extracting spatial information, even with random kernels, is sufficient to solve the MNIST task. The accuracy slightly degrades along the FA, DFA and DRTP algorithms, with a higher gap for shallow learning. When kernels are trained, BP provides the highest improvement compared to the error obtained with random kernels, followed by DRTP, while no significant change can be observed for FA and DFA. This is likely due to the fact that there is not enough parameter redundancy in convolutional layers to allow for an efficient training with feedback-alignment-based algorithms, which is commonly referred to as a *bottleneck effect* (see section 3). Indeed, the angle between the BP loss gradients and the feedback-alignment-based ones is roughly 90°, leading to random updates (Supplementary Figure 3). This improved performance of DRTP with trained kernels is thus unexpected. Regarding dropout, a positive impact is shown on BP, FA and DFA: a moderate dropout probability is beneficial for FC1 networks, while increasing it to 0.25 can be used for FC2 networks. Dropout has no positive impact for CONV networks, while it degrades the accuracy obtained with DRTP and shallow learning in all cases.

**Table 1:** Mean and standard deviation of the test error on the MNIST dataset over 10 trials.

**Network**		**BP (%)**	**FA (%)**	**DFA (%)**	**DRTP (%)**	**Shallow (%)**
FC1-500	DO 0.0	1.65 ± 0.06	1.71 ± 0.05	1.76 ± 0.05	4.61 ± 0.13	8.25 ± 0.09
	DO 0.1	1.59 ± 0.03	1.63 ± 0.05	1.68 ± 0.03	4.92 ± 0.13	9.17 ± 0.11
	DO 0.25	1.76 ± 0.05	1.74 ± 0.04	1.86 ± 0.03	5.75 ± 0.09	10.15 ± 0.11
FC1-1000	DO 0.0	1.57 ± 0.04	1.62 ± 0.05	1.67 ± 0.03	4.10 ± 0.07	7.92 ± 0.10
	DO 0.1	1.48 ± 0.03	1.55 ± 0.05	1.58 ± 0.05	4.31 ± 0.06	9.29 ± 0.12
	DO 0.25	1.54 ± 0.04	1.56 ± 0.02	1.63 ± 0.03	4.94 ± 0.06	10.01 ± 0.17
FC2-500	DO 0.0	1.46 ± 0.08	1.72 ± 0.04	1.69 ± 0.06	4.58 ± 0.09	8.25 ± 0.10
	DO 0.1	1.46 ± 0.04	1.51 ± 0.04	1.57 ± 0.06	5.00 ± 0.07	9.33 ± 0.09
	DO 0.25	1.38 ± 0.04	1.69 ± 0.02	1.52 ± 0.03	5.94 ± 0.06	11.01 ± 0.12
FC2-1000	DO 0.0	1.50 ± 0.09	1.57 ± 0.06	1.65 ± 0.07	4.00 ± 0.10	7.85 ± 0.09
	DO 0.1	1.46 ± 0.02	1.46 ± 0.03	1.57 ± 0.03	4.25 ± 0.06	8.73 ± 0.08
	DO 0.25	1.38 ± 0.03	1.50 ± 0.05	1.45 ± 0.03	5.05 ± 0.09	9.84 ± 0.05
CONV (random)	DO 0.0	1.21 ± 0.05	1.30 ± 0.06	1.25 ± 0.08	1.82 ± 0.11	2.83 ± 0.19
	DO 0.1	1.25 ± 0.03	1.33 ± 0.06	1.30 ± 0.06	2.06 ± 0.08	4.74 ± 0.30
	DO 0.25	1.29 ± 0.04	1.32 ± 0.06	1.33 ± 0.05	2.60 ± 0.14	6.49 ± 0.35
CONV (trained)	DO 0.0	0.93 ± 0.04	1.22 ± 0.06	1.31 ± 0.06	1.48 ± 0.15	
	DO 0.1	1.03 ± 0.04	1.27 ± 0.06	1.34 ± 0.06	1.50 ± 0.17	–
	DO 0.25	1.00 ± 0.03	1.29 ± 0.04	1.40 ± 0.06	1.81 ± 0.20	

*DO stands for dropout and indicates the dropout probability used in the fully-connected layers of both FC and CONV networks. The FC networks consist of one (FC1) or two (FC2) hidden layers comprising 500 or 1,000 tanh units, with an output fully-connected layer of 10 sigmoid units. The CONV network topology is as follows: a convolutional layer with 32 5×5 kernels, a stride of 1 and a padding of 2, a max-pooling layer with 2×2 kernels and a stride of 2, a fully-connected layer of 1,000 tanh units and an output fully-connected layer of 10 sigmoid units*.

#### 2.4.2. CIFAR-10

The results on the CIFAR-10 dataset are summarized in Table 2, highlighting conclusions similar to those already drawn for the MNIST dataset. Compared to BP, accuracy degrades along the FA, DFA and DRTP algorithms. The gap is higher for DRTP, yet it again compares favorably to shallow learning, demonstrating that DRTP also allows training hidden layers to learn CIFAR-10 image classification without feedback. For CONV networks, if kernels are trained, only BP is able to provide a significant advantage. Due to the bottleneck effect, FA only provides a slight improvement, while DFA and DRTP are negatively impacted. Regarding dropout, a moderate probability of 0.1 works fairly well for BP, FA, DFA and DRTP, while a higher probability of 0.25 rarely provides any advantage. Dropout always leads to an accuracy reduction for shallow learning. Finally, data augmentation (DA) improves the accuracy of all algorithms and is more effective than dropout.

**Table 2:** Mean and standard deviation of the test error on the CIFAR-10 dataset over 10 trials.

**Network**		**BP (%)**	**FA (%)**	**DFA (%)**	**DRTP (%)**	**Shallow (%)**
FC1-500	DO 0.0	48.45 ± 0.38	49.38 ± 0.22	49.62 ± 0.29	53.92 ± 0.23	58.83 ± 0.27
	DO 0.1	47.48 ± 0.39	48.94 ± 0.22	48.85 ± 0.23	53.77 ± 0.17	59.33 ± 0.17
	DO 0.25	47.80 ± 0.21	48.62 ± 0.23	48.65 ± 0.29	54.26 ± 0.16	60.44 ± 0.14
	DA	45.87 ± 0.22	47.11 ± 0.34	47.34 ± 0.26	52.73 ± 0.31	58.60 ± 0.20
FC1-1000	DO 0.0	47.52 ± 0.30	48.47 ± 0.18	48.44 ± 0.34	53.34 ± 0.10	57.91 ± 0.17
	DO 0.1	46.42 ± 0.28	47.72 ± 0.19	47.79 ± 0.31	53.15 ± 0.15	58.35 ± 0.24
	DO 0.25	46.21 ± 0.16	47.11 ± 0.18	47.11 ± 0.25	53.39 ± 0.15	59.20 ± 0.18
	DA	45.01 ± 0.33	46.15 ± 0.36	46.24 ± 0.32	51.87 ± 0.32	57.40 ± 0.24
FC2-500	DO 0.0	49.03 ± 0.22	50.66 ± 0.24	50.45 ± 0.36	53.41 ± 0.35	59.62 ± 0.34
	DO 0.1	48.32 ± 0.16	49.64 ± 0.23	49.58 ± 0.30	54.06 ± 0.46	60.34 ± 0.24
	DO 0.25	49.96 ± 0.18	50.80 ± 0.16	50.00 ± 0.13	54.57 ± 0.33	61.77 ± 0.18
	DA	46.62 ± 0.10	48.55 ± 0.25	48.75 ± 0.28	52.54 ± 0.34	58.99 ± 0.20
FC2-1000	DO 0.0	48.81 ± 0.22	49.87 ± 0.18	50.05 ± 0.21	52.68 ± 0.25	58.59 ± 0.14
	DO 0.1	46.58 ± 0.24	47.97 ± 0.18	48.68 ± 0.34	52.45 ± 0.15	59.12 ± 0.13
	DO 0.25	47.65 ± 0.16	48.82 ± 0.15	48.08 ± 0.14	53.29 ± 0.31	60.52 ± 0.14
	DA	46.05 ± 0.14	47.41 ± 0.27	47.90 ± 0.19	51.27 ± 0.21	57.90 ± 0.23
CONV (random)	DO 0.0	29.83 ± 0.25	30.27 ± 0.45	29.98 ± 0.30	32.65 ± 0.38	44.89 ± 0.67
	DO 0.1	29.49 ± 0.36	29.58 ± 0.33	29.44 ± 0.31	32.57 ± 0.34	48.38 ± 0.33
	DO 0.25	30.39 ± 0.32	30.55 ± 0.28	30.31 ± 0.35	33.90 ± 0.53	52.27 ± 0.34
	DA	27.87 ± 0.25	28.52 ± 0.40	28.46 ± 0.43	31.04 ± 0.45	44.23 ± 0.42
CONV (trained)	DO 0.0	25.31 ± 0.25	29.92 ± 0.26	31.38 ± 0.38	35.82 ± 0.59	–
	DO 0.1	27.12 ± 0.23	28.98 ± 0.36	30.56 ± 0.41	35.17 ± 0.91	
	DO 0.25	25.61 ± 0.23	28.95 ± 0.17	31.23 ± 0.38	35.51 ± 0.61	
	DA	25.27 ± 0.26	28.16 ± 0.45	29.49 ± 0.49	34.39 ± 0.64	

*DO stands for dropout and indicates the dropout probability used in the fully-connected layers of both FC and CONV networks. DA stands for data augmentation, which consists in horizontally flipping the training images. No dropout is used for DA. The FC networks consist of one (FC1) or two (FC2) hidden layers comprising 500 or 1,000 tanh units, with an output fully-connected layer of 10 sigmoid units. The CONV network topology is as follows: two convolutional layers with respectively 64 and 256 3×3 kernels, a stride and a padding of 1, both followed by a max-pooling layer with 2×2 kernels and a stride of 2, then two fully-connected layers of 1,000 tanh units and an output fully-connected layer of 10 sigmoid units*.

## 3. Discussion

While the backpropagation of error algorithm allowed taking artificial neural networks to outperform humans on complex datasets such as ImageNet (He et al., 2015), the key problems of *weight transport* and *update locking* highlight how aiming at breaking accuracy records on standard datasets has diverted attention from hardware efficiency considerations. While accuracy is the key driver for applications that can be backed by significant GPU and CPU resources, the development of decentralized adaptive smart sensors calls for keeping hardware requirements of learning algorithms to a minimum. Moreover, it has been shown that weight transport and update locking are not biologically plausible (Grossberg, 1987; Baldi et al., 2018), following from the non-locality in both weight and gradient information. Therefore, there is currently an increasing interest in releasing these constraints in order to achieve higher hardware efficiency and to understand the mechanisms that could underlie biological synaptic plasticity.

The proposed DRTP algorithm successfully addresses both the weight transport and the update locking problems, which has only been partially demonstrated in previously-proposed approaches. Indeed, the FA and DFA algorithms only address the weight transport problem (Lillicrap et al., 2016; Nøkland, 2016). The error locality approach still suffers from the weight transport problem in the local classifiers (Mostafa et al., 2018; Nøkland and Eidnes, 2019; Kaiser et al., 2020), while the synthetic gradients approach requires backpropagating gradient information from deeper layers in order to train the layerwise gradient predictors (Czarnecki et al., 2017; Jaderberg et al., 2017). Both the error locality and the synthetic gradients approaches also incur computational overhead by requiring the addition of side local networks for error or gradient prediction. On the contrary, DRTP is a strikingly simple rule that alleviates the two key BP issues by enabling each layer to be updated with local information as the forward evaluation proceeds. In order to estimate the layerwise loss gradients δ*y*_*k*_ for each layer, the only operation required by DRTP is a label-dependent random vector selection (Figure 1D). Despite the absence of dedicated feedback pathways, we demonstrated on the MNIST and CIFAR-10 datasets that DRTP allows training hidden layers at low computational and memory costs, thus highlighting its suitability for deployment in adaptive smart sensors at the edge and for embedded systems in general. In terms of floating-point operations (FLOPs), the overhead of DRTP weight updates is approximately equal to the cost of the forward pass, assuming that (i) the number of classes of the problem is negligible compared to the number of units in the hidden layers, which is typical of edge computing tasks, and (ii) the learning rate is embedded in the magnitude of the random connectivity matrices $B_{k}^{T}$. Doubling the computational cost of shallow-learning networks (i.e. doubling the numbers of hidden units or hidden layers) does not allow recovering their performance gap compared to DRTP-updated networks (Tables 1, 2). Even more importantly when considering dedicated hardware implementations for edge computing, the memory requirements should be minimized so as to fit the whole network topology into on-chip memory resources. Indeed, accesses to off-chip DRAM memory are three orders of magnitude more expensive energy-wise than a 32-bit FLOP (Horowitz, 2014). Therefore, as opposed to increasing the resources of shallow-trained networks, DRTP offers a low-overhead training algorithm operating on small network topologies, ideally suiting edge-computing hardware requirements. These claims are proven *in silico* in Frenkel et al. (2020), where implementing DRTP in an event-driven convolutional processor requires only 16.8–% power and 11.8–% silicon area overheads and allows demonstrating a favorable accuracy-power-area tradeoff compared to both on-chip online- and off-chip offline-trained conventional machine learning accelerators on the MNIST dataset.

By solving the weight transport and update locking problems, DRTP also releases key biological implausibility issues. Neurons in the brain separate forward and backward information in somatic and dendritic compartments, a property that is highlighted in the formulation of three-factor synaptic plasticity rules (Urbanczik and Senn, 2014): pre-synaptic and post-synaptic activities are modulated by a third factor corresponding to a local dendritic voltage. Lillicrap et al. (2016) build on the idea that a separate dendritic compartment integrates higher-order feedback and generates local teaching signals, where the errors could be viewed as a mismatch between expected and actual perceptions or actions. This aspect is further emphasized in the subsequent work of Guerguiev et al. (2017) when framing DFA as a spike-based three-factor learning rule. In the case of DRTP, compared to DFA, the error signal is replaced by the targets, which could correspond to a modulation that bypasses the actual perceptions or realized actions, relying only on predictions or intentions. Furthermore, DRTP could come in line with recent findings in cortical areas that reveal the existence of output-independent target signals in the dendritic instructive pathways of intermediate-layer neurons (Magee and Grienberger, 2020). Understanding the mechanisms of synaptic plasticity is critical in the field of neuromorphic engineering, which aims at porting biological computational principles to hardware toward higher energy efficiency (Thakur et al., 2018; Rajendran et al., 2019). However, even simple local bio-inspired learning rules such as spike-timing-dependent plasticity (STDP) (Bi and Poo, 1998) can lead to non-trivial hardware requirements, which currently hinders adaptive neuromorphic systems from reaching high-density large-scale integration (Frenkel et al., 2019b). While adaptations of STDP, such as spike-dependent synaptic plasticity (SDSP) (Brader et al., 2007), release most of the STDP hardware constraints, their training performance is currently not sufficient to support deployability of neuromorphic hardware for real-world scenarios (Frenkel et al., 2019b,c). A three-factor formulation of DRTP would release the update locking problem in the spike-based three-factor formulations of DFA (Guerguiev et al., 2017; Neftci et al., 2017), which currently imply memory and control overhead in their hardware implementations (Detorakis et al., 2018; Park et al., 2019). Porting DRTP to neuromorphic hardware is thus a natural next step.

While DRTP relaxes structural, memory and computational requirements toward decentralized hardware deployment, the accuracy degradation over DFA comes from the fact that only the error sign is taken into account, not its class-dependent magnitude. This could be mitigated by keeping track of the error magnitude over the last samples in order to modulate the layerwise learning rates, at the expense of releasing the purely feedforward nature of DRTP. A learning rate scheduler could also be used. The DRTP algorithm was derived specifically for classification problems with sigmoid/softmax output units and a binary/categorical cross-entropy loss, yet hidden layer activations also play a key role in the learning dynamics of DRTP. As the estimated loss gradients δ*y*_*k*_ computed from the targets have a constant sign and magnitude, the weights updates only change due to the previous layer outputs and the derivative of the activation function, as training progresses. When using activation functions such as tanh in the hidden layers, the network stops learning thanks to the activation function derivative, whose value vanishes as its input argument moves away from zero. This mechanism specific to DRTP is highlighted in Supplementary Figures 4–6 and could be exploited to generate networks whose activations can be binarized during inference, which we will investigate in future work. In return, only activation functions presenting this saturation property are expected to lead to satisfying performance when used in conjunction with DRTP, which for example excludes ReLU activations.

Finally, as for all other feedback-alignment-based algorithms, DRTP only slightly improves or even degrades the accuracy when applied to convolutional layers. Convolutional layers do not provide the parameter redundancy that can be found in fully-connected layers, a *bottleneck effect* that was first highlighted for FA (Lillicrap et al., 2016) and has recently been studied for DFA (Launay et al., 2019). Nevertheless, other training algorithms based either on a greedy layerwise learning (Belilovsky et al., 2019) or on the alignment with local targets (Ororbia and Mali, 2019) have proven to be successful in training convolutional layers at the expense of only partially solving the update locking problem. Indeed, the training algorithm proposed in Belilovsky et al. (2019) still suffers from update locking in the layerwise auxiliary networks while the one proposed in Ororbia and Mali (2019) relies on the backpropagation of the output error to compute the layerwise targets. If fixed random convolutional layers do not meet the performance requirements of the target application, a combination of DRTP for fully-connected layers together with error locality or synthetic gradients approaches for convolutional layers can be considered. This granularity in the selection of learning mechanisms, trading off accuracy and hardware efficiency, comes in accordance with the wide spectrum of plasticity mechanisms that are believed to operate in the brain (Zenke et al., 2015).

## 4. Materials and Methods

The training on both the synthetic regression and classification tasks and the MNIST and CIFAR-10 datasets has been carried out with PyTorch (Paszke et al., 2017), one of the numerous Python frameworks supporting deep learning. In all experiments, the reported update angles between feedback-alignment-based algorithms and BP were generated at each update step, where the BP update values were computed solely to assess the evolution of the alignment angle over the update steps carried out by FA, DFA, sDFA or DRTP.

### 4.1. Regression

The examples in the training and test sets are denoted as (x, y^*^). The 10-dimensional target vectors y^*^ are generated using $y_{j}^{*}=T_{j}\left(x\right)=\text{cos}\left(\bar{x}+\phi_{j}\right)$, where ϕ_*j*_ = −π/2+*jπ*/9 for *j* ∈ [0, 9] and *j* ∈ ℕ_0_. $\bar{x}$ denotes the mean of *x*, a 256-dimensional vector whose entries are initialized from a normal distribution with a mean sampled from a uniform distribution between −π and π and with a unit variance. The training and test sets respectively contain 5k and 1k examples. The trained network has a 256-100-100-10 topology with tanh hidden and output units, whose forward weights are drawn from a He uniform distribution (He et al., 2015) and are zero-initialized for feedback-alignment-based algorithms. The random connectivity matrices of feedback-alignment-based algorithms are also drawn from He uniform distributions. The weights are updated after each minibatch of 50 examples, and the network is trained for 500 epochs with a fixed learning rate η = 5 × 10^−4^ for all training algorithms. As this is a regression task, the loss function is the mean squared error. The losses on the training and test sets and the alignment angles with BP updates are monitored every 1k samples. The experiment is repeated 10 times for each training algorithm, with different network initializations for each experiment run.

### 4.2. Synthetic Data Classification

The examples in the training and test sets are generated using the make_classification function from the Python library sklearn (Pedregosa et al., 2011). The main inputs required by this function are the number of samples to be generated, the number of features *n* in the input vectors *x*, the number of informative features *n*_*inf*_ among the input vectors, the number of classes, the number of clusters per class and a factor class_sep which conditions the class separation. In this work, we have used *n* = 256 and *n*_*inf*_ = 128, ten classes, five clusters per class and class_sep = 4.5. Using this set of parameters, the make_classification function then generates examples by creating for each class clusters of points normally distributed about the vertices of an *n*_*inf*_-dimensional hypercube. The remaining features are filled with normally-distributed random noise. The generated examples are then separated into training and test sets of 25k and 5k examples, respectively. The trained network has a 256-500-500-10 topology with tanh hidden units and sigmoid output units. The forward and backward weights initialization, as well as the forward weight updates, are performed as for regression. The loss function is the binary cross-entropy loss. The network is trained for 500 epochs with a fixed learning rate η = 5 × 10^−4^. The losses on the training and test sets and the alignment angles with BP updates are monitored every 2.5k samples. For each training algorithm, the experiment is repeated 10 times with different network initializations.

### 4.3. MNIST and CIFAR-10 Images Classification

A fixed learning rate is selected based on a grid search for each training algorithm, dataset and network type (Table 3). For both the MNIST and CIFAR-10 experiments, the chosen optimizer is Adam with default parameters. A sigmoid output layer and a binary cross-entropy loss are used for all training algorithms. The entries of the forward weight matrices *W*_*k*_ are initialized with a He uniform distribution, as well as the entries of the fixed random connectivity matrices *B*_*k*_ of feedback-alignment-based algorithms. When used, dropout is applied with the same probability to all fully-connected layers. For MNIST, the networks are trained for 100 epochs with a minibatch size of 60. The CONV network topology consists of a convolutional layer with 32 5×5 kernels, a stride of 1 and a padding of 2, a max-pooling layer with 2×2 kernels and a stride of 2, a fully-connected layer of 1,000 tanh units and an output fully-connected layer of 10 units. For CIFAR-10, a minibatch size of 100 is used and early stopping is applied, with a maximum of 200 epochs. The CONV network topology consists of two convolutional layers with respectively 64 and 256 3×3 kernels, a stride and a padding of 1, both followed by a max-pooling layer with 2×2 kernels and a stride of 2, then two fully-connected layers of 1,000 tanh units and an output fully-connected layer of 10 units. For all experiments, the test error is averaged over the last 10 epochs of training. The results reported in Tables 1, 2 and Supplementary Tables 1, 2 are the mean and standard deviation over 10 trials.

**Table 3:** The learning rate values for the MNIST and CIFAR-10 datasets are selected based on a grid search.

**Dataset**	**Network**	**BP**	**FA**	**DFA**	**sDFA**	**DRTP**	**Shallow**
MNIST	FC1	1.5 × 10^−4^	5 × 10^−4^	1.5 × 10^−4^	5 × 10^−4^	1.5 × 10^−4^	1.5 × 10^−2^
	FC2	5 × 10^−4^	1.5 × 10^−4^	5 × 10^−4^	5 × 10^−4^	1.5 × 10^−4^	5 × 10^−3^
	CONV (rand.)	5 × 10^−5^	1.5 × 10^−4^	5 × 10^−5^	5 × 10^−4^	5 × 10^−4^	5 × 10^−3^
	CONV (train.)	5 × 10^−4^	5 × 10^−5^	5 × 10^−5^	1.5 × 10^−4^	1.5 × 10^−4^	–
CIFAR-10	FC1	1.5 × 10^−5^	1.5 × 10^−5^	1.5 × 10^−5^	5 × 10^−5^	1.5 × 10^−4^	1.5 × 10^−4^
	FC2	5 × 10^−6^	5 × 10^−6^	5 × 10^−6^	5 × 10^−5^	5 × 10^−5^	5 × 10^−4^
	CONV (rand.)	5 × 10^−6^	5 × 10^−6^	5 × 10^−6^	1.5 × 10^−4^	1.5 × 10^−4^	1.5 × 10^−3^
	CONV (train.)	1.5 × 10^−4^	5 × 10^−6^	5 × 10^−6^	1.5 × 10^−5^	5 × 10^−5^	–

*A different learning rate is selected for each training algorithm, dataset and network type*.

## Data Availability Statement

Publicly available datasets were analyzed in this study. The data can be found at: http://yann.lecun.com/exdb/mnist/ and https://www.cs.toronto.edu/~kriz/cifar.html.

## Code Availability

The PyTorch code allowing to reproduce all results in this study is available open source under the Apache 2.0 license at https://github.com/ChFrenkel/DirectRandomTargetProjection.

## Author Contributions

CF developed the main idea. CF and ML derived the mathematical proofs and worked on the simulation experiments. CF, ML, and DB wrote the paper. All authors contributed to the article and approved the submitted version.

## Conflict of Interest

The authors declare that the research was conducted in the absence of any commercial or financial relationships that could be construed as a potential conflict of interest.

## References

[B1] Amodei D. Ananthanarayanan S. Anubhai R. Bai J. Battenberg E. Case C. (2016). “Deep speech 2: end-to-end speech recognition in English and Mandarin,” in Proceedings of the 33rd International Conference on Machine Learning (New York, NY), 173–182.

[B2] Baldi P. Sadowski P. Lu Z. (2018). Learning in the machine: random backpropagation and the deep learning channel. Artif. Intell. 260, 1–35. 10.1016/j.artint.2018.03.003 29731511 PMC5931406

[B3] Bartunov S. Santoro A. Richards B. Marris L. Hinton G. E. Lillicrap T. (2018). “Assessing the scalability of biologically-motivated deep learning algorithms and architectures,” in Advances in Neural Information Processing Systems, eds Bengio S. Wallach H. Larochelle H. Grauman K. Cesa-Bianchi N. Garnett R. (Montreal, QC: Curran Associates, Inc.), 9368–9378.

[B4] Bassett D. S. Bullmore E. T. (2006). Small-world brain networks. Neuroscientist 12, 512–523. 10.1177/1073858406293182 17079517

[B5] Belilovsky E. Eickenberg M. Oyallon E. (2019). Decoupled greedy learning of CNNs. arXiv preprint arXiv:1901.08164.

[B6] Bi G.-Q. Poo M.-M. (1998). Synaptic modifications in cultured hippocampal neurons: dependence on spike timing, synaptic strength, and postsynaptic cell type. J. Neurosci. 18, 10464–10472. 10.1523/JNEUROSCI.18-24-10464.1998 9852584 PMC6793365

[B7] Bol D. de Streel G. Flandre D. (2015). “Can we connect trillions of IoT sensors in a sustainable way? A technology/circuit perspective,” in 2015 IEEE SOI-3D-Subthreshold Microelectronics Technology Unified Conference (S3S) (Sonoma Valley, CA: IEEE), 1–3. 10.1109/S3S.2015.7333500

[B8] Brader J. M. Senn W. Fusi S. (2007). Learning real-world stimuli in a neural network with spike-driven synaptic dynamics. Neural Comput. 19, 2881–2912. 10.1162/neco.2007.19.11.2881 17883345

[B9] Crafton B. West M. Basnet P. Vogel E. Raychowdhury A. (2019). “Local learning in RRAM neural networks with sparse direct feedback alignment,” in 2019 IEEE/ACM International Symposium on Low Power Electronics and Design (ISLPED) (Lausanne: IEEE), 1–6. 10.1109/ISLPED.2019.8824820

[B10] Czarnecki W. M. Świrszcz G. Jaderberg M. Osindero S. Vinyals O. Kavukcuoglu K. (2017). “Understanding synthetic gradients and decoupled neural interfaces,” in Proceedings of the 34th International Conference on Machine Learning (Sydney, NSW), 904–912.

[B11] Deng J. Dong W. Socher R. Li L. J. Li K. Fei-Fei L. (2009). “ImageNet: A large-scale hierarchical image database,” in 2009 IEEE Conference on Computer Vision and Pattern Recognition (CVPR) (Miami, FL), 248–255. 10.1109/CVPR.2009.5206848

[B12] Detorakis G. Sheik S. Augustine C. Paul S. Pedroni B. U. Dutt N.. (2018). Neural and synaptic array transceiver: a brain-inspired computing framework for embedded learning. Front. Neurosci. 12:583. 10.3389/fnins.2018.00583 30210274 PMC6123384

[B13] Frenkel C. Lefebvre M. Bol D. (2019a). Learning without feedback: direct random target projection as a feedback-alignment algorithm with layerwise feedforward training. arXiv preprint arXiv:1909.01311.

[B14] Frenkel C. Lefebvre M. Legat J.-D. Bol D. (2019b). A 0.086-mm^2^ 12.7-pJ/SOP 64k-synapse 256-neuron online-learning digital spiking neuromorphic processor in 28-nm CMOS. IEEE Trans. Biomed. Circ. Syst. 13, 145–158. 10.1109/TBCAS.2018.2880425 30418919

[B15] Frenkel C. Legat J.-D. Bol D. (2019c). MorphIC: a 65-nm 738k-synapse/mm^2^ quad-core binary-weight digital neuromorphic processor with stochastic spike-driven online learning. IEEE Trans. Biomed. Circ. Syst. 13, 999–1010. 10.1109/TBCAS.2019.2928793 31329562

[B16] Frenkel C. Legat J.-D. Bol D. (2020). “A 28-nm convolutional neuromorphic processor enabling online learning with spike-based retinas,” in 2020 IEEE International Symposium on Circuits and Systems (ISCAS) (Sevilla: IEEE). 10.1109/ISCAS45731.2020.9180440

[B17] Grossberg S. (1987). Competitive learning: From interactive activation to adaptive resonance. Cogn. Sci. 11, 23–63. 10.1111/j.1551-6708.1987.tb00862.x

[B18] Guerguiev J. Lillicrap T. P. Richards B. A. (2017). Towards deep learning with segregated dendrites. eLife 6:e22901. 10.7554/eLife.22901 29205151 PMC5716677

[B19] He K. Zhang X. Ren S. Sun J. (2015). “Delving deep into rectifiers: Surpassing human-level performance on ImageNet classification,” in Proceedings of the IEEE International Conference on Computer Vision (Santiago), 1026–1034. 10.1109/ICCV.2015.123

[B20] He K. Zhang X. Ren S. Sun J. (2016). “Deep residual learning for image recognition,” in Proceedings of the IEEE Conference on Computer Vision and Pattern Recognition (Las Vegas, NV), 770–778. 10.1109/CVPR.2016.90

[B21] Hinton G. E. Deng L. Yu D. Dahl G. E. Mohamed A.-R. Jaitly N. (2012). Deep neural networks for acoustic modeling in speech recognition: the shared views of four research groups. IEEE Signal Process. Mag. 29, 82–97. 10.1109/MSP.2012.2205597

[B22] Horowitz M. (2014). “Computing's energy problem (and what we can do about it),” in IEEE International Solid-State Circuits Conference Digest of Technical Papers (ISSCC) (San Francisco, CA: IEEE), 10–14. 10.1109/ISSCC.2014.6757323

[B23] Ioffe S. Szegedy C. (2015). Batch normalization: accelerating deep network training by reducing internal covariate shift. arXiv preprint arXiv:1502.03167.

[B24] Jaderberg M. Czarnecki W. M. Osindero S. Vinyals O. Graves A. Silver D. (2017). “Decoupled neural interfaces using synthetic gradients,' in Proceedings of the 34th International Conference on Machine Learning (Sydney, NSW), 1627–1635.

[B25] Kaiser J. Mostafa H. Neftci E. (2020). Synaptic plasticity dynamics for deep continuous local learning (DECOLLE). Front. Neurosci. 14:424. 10.3389/fnins.2020.00424 32477050 PMC7235446

[B26] Krizhevsky A. Hinton G. E. (2009). Learning multiple layers of features from tiny images.

[B27] Krizhevsky A. Sutskever I. Hinton G. E. (2012). “ImageNet classification with deep convolutional neural networks,” in Advances in Neural Information Processing Systems, eds Pereira F. Burges C. J. C. Bottou L. Weinberger K. Q. (Lake Tahoe, NV: Curran Associates, Inc.), 1097–1105.

[B28] Launay J. Poli I. Krzakala F. (2019). Principled training of neural networks with direct feedback alignment. arXiv preprint arXiv:1906.04554.

[B29] LeCun Y. Bengio Y. Hinton G. E. (2015). Deep learning. Nature 521, 436–444. 10.1038/nature14539 26017442

[B30] LeCun Y. Cortes C. Burges C. J. (1998). The MNIST database of handwritten digits.

[B31] Lee D.-H. Zhang S. Fischer A. Bengio Y. (2015). “Difference target propagation,” in Joint European Conference on Machine Learning and Knowledge Discovery in Databases (Würzburg), 498–515. 10.1007/978-3-319-23528-8_31

[B32] Liao Q. Leibo J. Z. Poggio T. (2016). “How important is weight symmetry in backpropagation?” in Thirtieth AAAI Conference on Artificial Intelligence (Phoenix, AZ).

[B33] Lillicrap T. P. Cownden D. Tweed D. B. Akerman C. J. (2016). Random synaptic feedback weights support error backpropagation for deep learning. Nat. Commun. 7, 1–10. 10.1038/ncomms13276 27824044 PMC5105169

[B34] Magee J. C. Grienberger C. (2020). Synaptic plasticity forms and functions. Annu. Rev. Neurosci. 43, 95–117. 10.1146/annurev-neuro-090919-022842 32075520

[B35] Milde M. B. Blum H. Dietmüller A. Sumislawska D. Conradt J. Indiveri G.. (2017). Obstacle avoidance and target acquisition for robot navigation using a mixed signal analog/digital neuromorphic processing system. Front. Neurorobot. 11:28. 10.3389/fnbot.2017.00028 28747883 PMC5507184

[B36] Minsky M. (1961). Steps toward artificial intelligence. Proc. IRE 49, 8–30. 10.1109/JRPROC.1961.287775

[B37] Mostafa H. Ramesh V. Cauwenberghs G. (2018). Deep supervised learning using local errors. Front. Neurosci. 12:608. 10.3389/fnins.2018.00608 30233295 PMC6127296

[B38] Neftci E. O. Augustine C. Paul S. Detorakis G. (2017). Event-driven random back-propagation: enabling neuromorphic deep learning machines. Front. Neurosci. 11:324. 10.3389/fnins.2017.00324 28680387 PMC5478701

[B39] Nøkland A. (2016). “Direct feedback alignment provides learning in deep neural networks,” in Advances in Neural Information Processing Systems, eds Lee D. Sugiyama M. Luxburg U. Guyon I. Garnett R. (Barcelona: Curran Associates, Inc.) 1037–1045.

[B40] Nøkland A. Eidnes L. H. (2019). “Training neural networks with local error signals,” in Proceedings of the 36th International Conference on Machine Learning (Long Beach, CA), 4839–4850.

[B41] Ororbia A. G. Mali A. (2019). “Biologically motivated algorithms for propagating local target representations,” in Proceedings of the AAAI Conference on Artificial Intelligence (Honolulu, HI), 4651–4658. 10.1609/aaai.v33i01.33014651

[B42] Park J. Lee J. Jeon D. (2019). A 65-nm neuromorphic image classification processor with energy-efficient training through direct spike-only feedback. IEEE J. Solid State Circ. 55, 108–119. 10.1109/JSSC.2019.2942367

[B43] Paszke A. Gross S. Chintala S. Chanan G. Yang E. DeVito Z. (2017). “Automatic differentiation in PyTorch,” in Proceedings of the 31st Conference of Neural Information Processing Systems (NIPS 2017) (Long Beach, CA).

[B44] Pedregosa F. Varoquaux G. Gramfort A. Michel V. Thirion B. Grisel O. (2011). Scikit-learn: machine learning in Python. J. Mach. Learn. Res. 12, 2825–2830.

[B45] Rajendran B. Sebastian A. Schmuker M. Srinivasa N. Eleftheriou E. (2019). Low-power neuromorphic hardware for signal processing applications: a review of architectural and system-level design approaches. IEEE Signal Process. Mag. 36, 97–110. 10.1109/MSP.2019.2933719

[B46] Rosenblatt F. (1962). Principles of Neurodynamics: Perceptions and the Theory of Brain Mechanisms. Washington, DC: Spartan 10.21236/AD0256582

[B47] Rumelhart D. E. Hinton G. E. Williams R. J. (1986). Learning representations by back-propagating errors. Nature 323, 533–536. 10.1038/323533a0

[B48] Thakur C. S. Molin J. L. Cauwenberghs G. Indiveri G. Kumar K. Qiao N.. (2018). Large-scale neuromorphic spiking array processors: a quest to mimic the brain. Front. Neurosci. 12:891. 10.3389/fnins.2018.00891 30559644 PMC6287454

[B49] Urbanczik R. Senn W. (2014). Learning by the dendritic prediction of somatic spiking. Neuron 81, 521–528. 10.1016/j.neuron.2013.11.030 24507189

[B50] Zenke F. Agnes E. J. Gerstner W. (2015). Diverse synaptic plasticity mechanisms orchestrated to form and retrieve memories in spiking neural networks. Nat. Commun. 6, 1–13. 10.1038/ncomms7922 PMC4411307 25897632

