# Supplementary material for: Learning Without Feedback: Fixed Random Learning Signals Allow for Feedforward Training of Deep Neural Networks
Source: Front Neurosci. 2021 Feb 10;15:629892. doi: 10.3389/fnins.2021.629892 (PMC7902857; doi:10.3389/fnins.2021.629892)
Supplement: Supplementary file 1 [file Data_Sheet_1.pdf]

Supplementary Material

Learning without feedback: Fixed random learning signals allow for feedforward training of deep neural networks

Charlotte Frenkel, Martin Lefebvre and David Bol

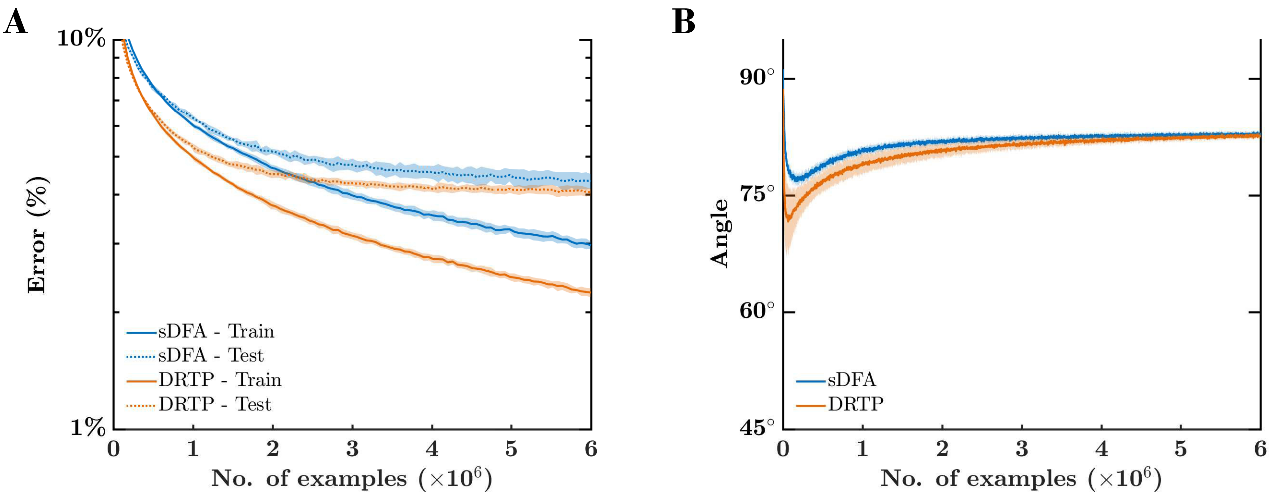

Supplementary Figure 1. DRTP outperforms sDFA on the MNIST dataset. Both figures are with error bars of one standard deviation over 10 runs. The training and test errors are measured after each epoch, while the angle is measured after each minibatch of 60 examples. Both training methods use Adam with a fixed learning rate of 1.5×10^{-4}. **(A)** A 784-1000-10 network with tanh hidden units and sigmoid output units is trained to classify MNIST handwritten digits with the sDFA and DRTP algorithms. On average, the error on the training set reaches 2.97% for sDFA and 2.24% for DRTP, while the error on the test set reaches 4.33% for sDFA and 4.05% for DRTP after 100 epochs. **(B)** While the loss gradients δy_k estimated by both sDFA and DRTP are within 90° of the ones prescribed by BP, the alignment angle is initially better for DRTP than for sDFA. The gap vanishes as the training progresses.

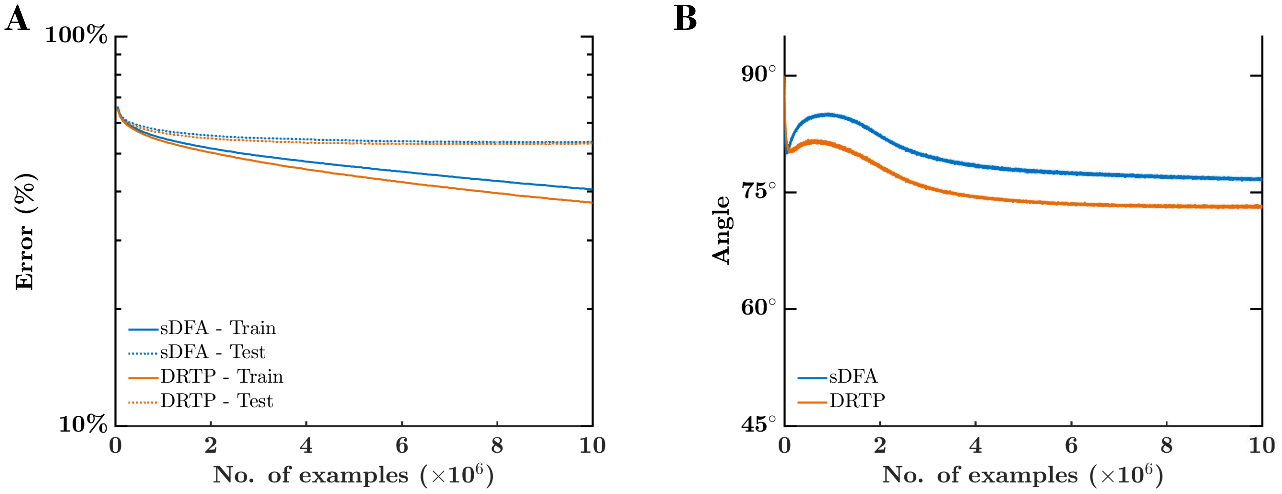

Supplementary Figure 2. DRTP outperforms sDFA on the CIFAR-10 dataset. Both figures are with error bars of one standard deviation over 10 runs. The training and test errors are measured after each epoch, while the angle is measured after each minibatch of 100 examples. Both training methods use Adam with a fixed learning rate of $5{\times}10^{-5}$. **(A)** A 3072-1000-10 network with tanh hidden units and sigmoid output units is trained to classify CIFAR-10 images with the sDFA and DRTP algorithms. On average, the error on the training set reaches 40.74% for sDFA and 37.39% for DRTP, while the error on the test set reaches 53.53% for sDFA and 53.12% for DRTP after 200 epochs. No early stopping was applied. **(B)** While the loss gradients δy_k estimated by both sDFA and DRTP are within 90° of the ones prescribed by BP, the alignment angle is approximately 3.40° better for DRTP than for sDFA.

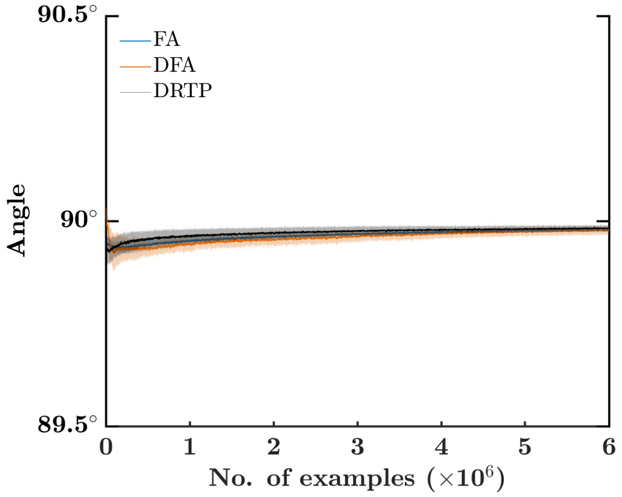

Supplementary Figure 3. Updates to the convolutional layer weights prescribed by feedback-alignment-based algorithms are random due to a 90°-alignment with the BP loss gradients δy_k. A convolutional network is trained on the MNIST dataset with FA, DFA and DRTP. The network topology and training parameters are identical to those used for the trained CONV network. Error bars are one standard deviation over 10 runs, the angle is measured after each minibatch of 60 examples. Angles have been smoothed by an exponentially-weighted moving average filter with a momentum coefficient of 0.95.

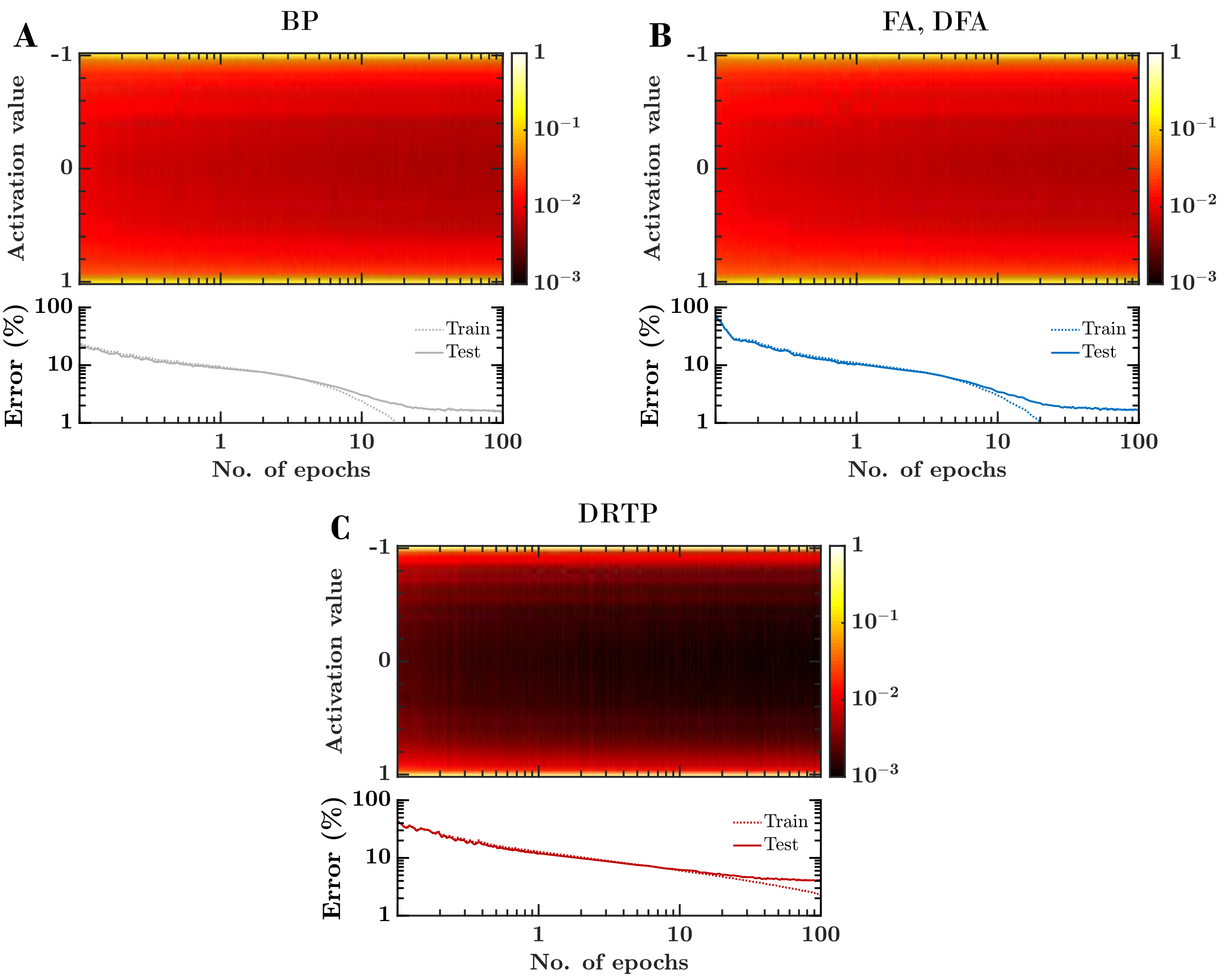

Supplementary Figure 4. On the MNIST dataset, DRTP leads to a distribution of the activation values in the hidden layer that is more heavily skewed towards ±1 than BP, FA and DFA. A 784-1000-10 network with tanh hidden units and sigmoid output units is trained to classify MNIST images with the BP (**A**), FA/DFA (**B**) and DRTP (**C**) algorithms, where the FA and DFA algorithms are equivalent for single-hidden-layer networks. The network training relies on the Adam optimizer with a binary cross-entropy loss and a fixed learning rate of 1.5×10^{-4}, the training and test errors are measured after each minibatch during the first epoch and then after each epoch during the rest of the training. To estimate the probability density function of the activations, their values are monitored for 100 different examples in 100 successive minibatches over the course of training. The estimated probability density function hints at a different learning mechanism for DRTP: as the distribution of the activation values in the hidden layer is more heavily skewed towards ±1, the vanishing value of the tanh activation function derivative in this region leads the network to stop learning.

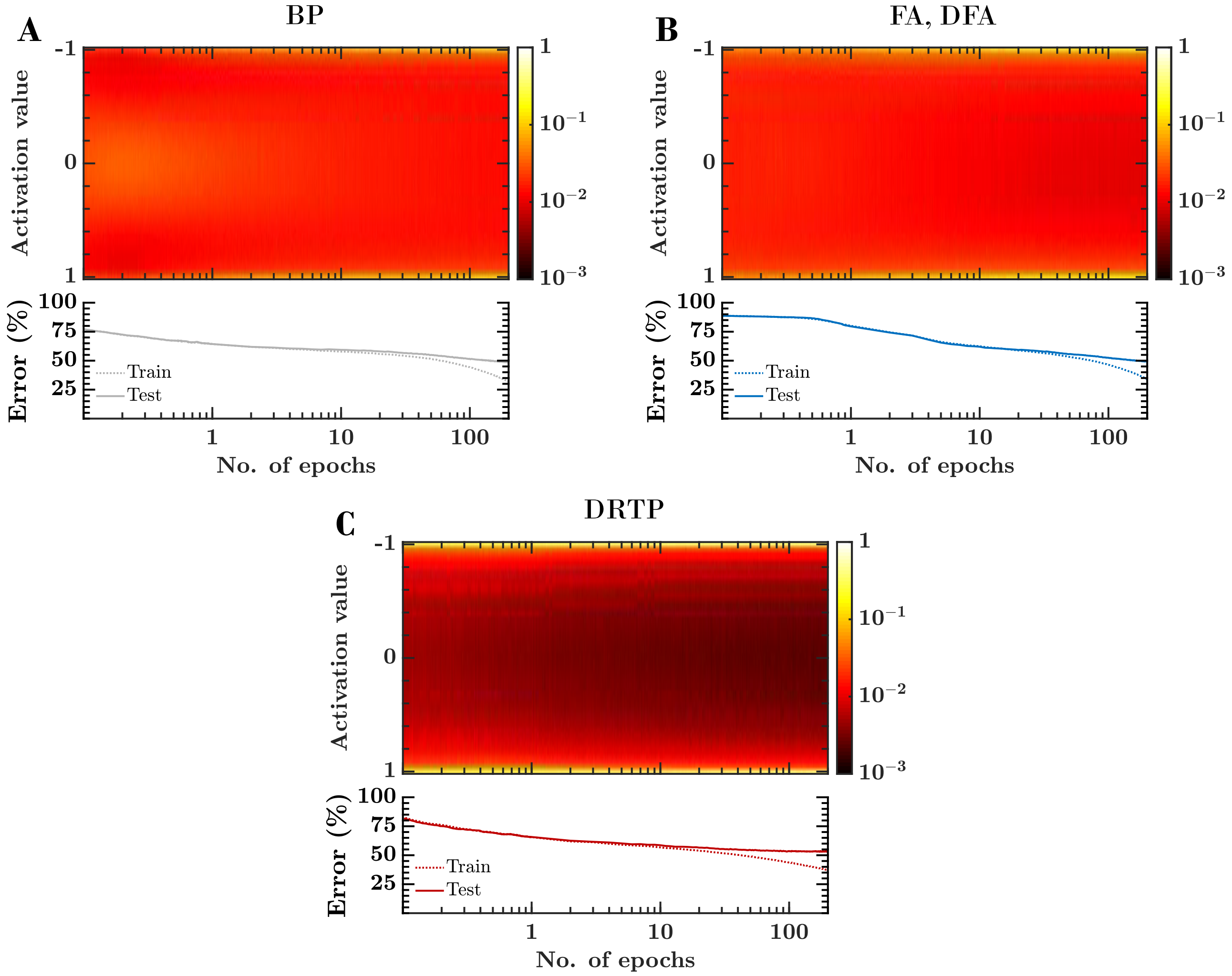

Supplementary Figure 5. On the CIFAR-10 dataset, DRTP leads to a distribution of the activation values in the hidden layer that is more heavily skewed towards ± 1 than BP, FA and DFA. A 3072-1000-10 network with tanh hidden units and sigmoid output units is trained to classify CIFAR-10 images with the BP (**A**), FA/DFA (**B**) and DRTP (**C**) algorithms. The network training relies on the Adam optimizer with a binary cross-entropy loss and a fixed learning rate of 5×10^{-6} for BP and FA/DFA, and 5×10^{-5} for DRTP, as per Table 3 in the main text. Other experimental conditions are similar to the ones used in Supplementary Fig. 4. Similarly to the experiments on the MNIST dataset, the distribution of the activation values in the hidden layer is more heavily skewed towards ± 1 for DRTP, which hints at a stop learning mechanism through the vanishing value of the tanh activation function derivative in this region.

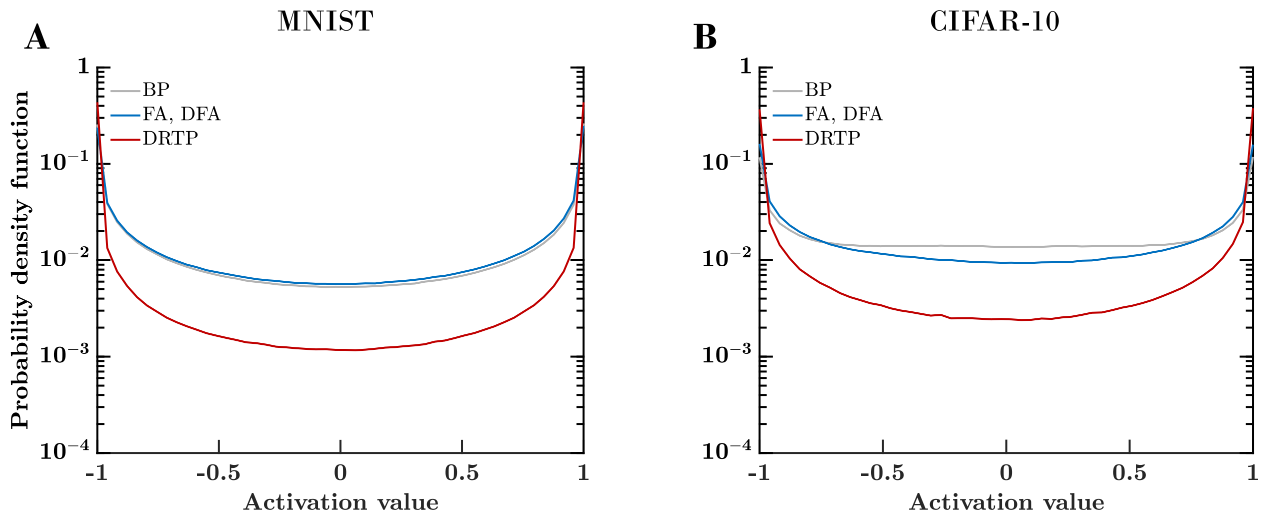

Supplementary Figure 6. The estimated probability density function of the activation values at the end of training exhibits a more pronounced skewing towards ±1 for DRTP than for BP, FA and DFA. Single-hidden-layer fully-connected networks are trained to classify images from the MNIST (**A**) and CIFAR-10 (**B**) datasets, with the experimental conditions described in Supplementary Figs. 4 and 5.

Supplementary Table 1. Comparison of the sDFA and DRTP training algorithms on the MNIST dataset, illustrating that DRTP systematically outperforms sDFA. The mean and the standard deviation of the test error over 10 trials are provided. The network definitions and conditions are identical to those of Table **1** in the main text. The learning rates are summarized in Table **3** in the main text.

Network		sDFA	DRTP
FC1-500	DO 0.0	4.74±0.15%	4.61±0.13%
	DO 0.1	5.10±0.13%	4.92±0.13%
	DO 0.25	6.06±0.10%	5.75±0.09%
FC1-1000	DO 0.0	4.22±0.11%	4.10±0.07%
	DO 0.1	4.42±0.12%	4.31±0.06%
	DO 0.25	5.23±0.12%	4.94±0.06%
FC2-500	DO 0.0	4.78±0.11%	4.58±0.09%
	DO 0.1	5.16±0.13%	5.00±0.07%
	DO 0.25	6.13±0.10%	5.94±0.06%
FC2-1000	DO 0.0	4.24±0.09%	4.00±0.10%
	DO 0.1	4.51±0.12%	4.25±0.06%
	DO 0.25	5.39±0.05%	5.05±0.09%
CONV (random)	DO 0.0	1.88±0.10%	1.82±0.11%
	DO 0.1	2.17±0.13%	2.06±0.08%
	DO 0.25	2.80±0.17%	2.60±0.14%
CONV (trained)	DO 0.0	1.69±0.10%	1.48±0.15%
	DO 0.1	1.83±0.11%	1.50±0.17%
	DO 0.25	2.20±0.15%	1.81±0.20%

Supplementary Table 2. Comparison of the sDFA and DRTP training algorithms on the CIFAR-10 dataset, illustrating that DRTP systematically outperforms sDFA. The mean and the standard deviation of the test error over 10 trials are provided. The network definitions and conditions are identical to those of Table **2** in the main text. The learning rates are summarized in Table **3** in the main text.

Network		sDFA	DRTP
FC1-500	DO 0.0	54.80±0.29%	53.92±0.23%
	DO 0.1	54.79±0.24%	53.77±0.17%
	DO 0.25	55.48±0.27%	54.26±0.16%
	DA	53.83±0.32%	52.73±0.31%
FC1-1000	DO 0.0	53.73±0.33%	53.34±0.10%
	DO 0.1	53.92±0.31%	53.15±0.15%
	DO 0.25	54.60±0.38%	53.39±0.15%
	DA	52.95±0.32%	51.87±0.32%
FC2-500	DO 0.0	54.75±0.26%	53.41±0.35%
	DO 0.1	55.35±0.38%	54.06±0.46%
	DO 0.25	55.81±0.37%	54.57±0.33%
	DA	53.85±0.34%	52.54±0.34%
FC2-1000	DO 0.0	53.78±0.24%	52.68±0.25%
	DO 0.1	53.87±0.49%	52.45±0.15%
	DO 0.25	54.87±0.43%	53.29±0.31%
	DA	52.59±0.20%	51.27±0.21%
CONV (random)	DO 0.0	33.08±0.31%	32.65±0.38%
	DO 0.1	33.04±0.42%	32.57±0.34%
	DO 0.25	34.71±0.37%	33.90±0.53%
	DA	31.52±0.25%	31.04±0.45%
CONV (trained)	DO 0.0	38.69±0.78%	35.82±0.59%
	DO 0.1	39.23±0.82%	35.17±0.91%
	DO 0.25	40.08±1.03%	35.51±0.61%
	DA	38.43±0.86%	34.39±0.64%

SUPPLEMENTARY NOTE 1 DETAILED PROOF OF ALIGNMENT BETWEEN THE BP AND DRTP MODULATORY SIGNALS

This full version of the alignment proof between the BP and DRTP modulatory signals is derived for a neural network composed of linear hidden layers (Figure **4** in the main text) and a single training example (x, c^*), where x is the input data sample and c^* the label. The C-dimensional target vector y^* corresponds to the one-hot encoding of c^*, where C is the number of classes. Our developments build on the alignment proof of Lillicrap et al. (2016), which demonstrates that the FA and BP modulatory signals are within 90° of each other in the case of a single linear hidden layer, a linear output layer and a mean squared error loss. In the framework of classification problems, we extend this proof for the case of DRTP and to an arbitrary number of linear hidden layers, a nonlinear output layer of sigmoid/softmax units and a binary/categorical cross-entropy loss.

Network dynamics. The output of the k-th linear hidden layer is given by

$$y_k = z_k = W_k y_{k-1} \quad \text{for } k \in [1, K-1],$$

where K is the number of layers and $y_0 = x$ is the input vector. Note that the bias vector b_k is omitted without loss of generality. The output layer is described by

$$\begin{aligned} z_K &= W_K y_{K-1}, \\ y_K &= \sigma\left(z_K\right), \end{aligned}$$

where $\sigma(\cdot)$ is either the sigmoid or the softmax activation function. The loss function $J(\cdot)$ is either the binary cross-entropy (BCE) loss for sigmoid output units or the categorical cross-entropy (CCE) loss for softmax output units, computed over the C output classes:

$$\begin{aligned} J_{\text{BCE}}(y_K, y^*) &= -\frac{1}{C}\sum_{c=1}^{C}\Big(y_c^*\log\left(y_{Kc}\right) + (1-y_c^*)\log\left(1-y_{Kc}\right)\Big), \\ J_{\text{CCE}}(y_K, y^*) &= -\frac{1}{C}\sum_{c=1}^{C}\Big(y_c^*\log\left(y_{Kc}\right)\Big). \end{aligned}$$

The network is trained with stochastic gradient descent. In the output layer, the weight updates of both BP and DRTP follow

$$W_{K,ji} \leftarrow W_{K,ji} - \eta\sum_{l=1}^{C}\frac{\partial J}{\partial z_{Kl}}\frac{\partial z_{Kl}}{\partial W_{K,ji}}$$

where $i, j \in \mathbb{N}$ are indices corresponding respectively to the columns and rows of the output layer weight matrix. For both sigmoid and softmax output units, the factors in this update can be computed as

$$\begin{aligned} \frac{\partial J}{\partial z_{Kl}} &= \sum_{c=1}^{C}\frac{\partial J}{\partial y_{Kc}}\frac{\partial y_{Kc}}{\partial z_{Kl}}, \\ \frac{\partial z_{Kl}}{\partial W_{K,ji}} &= \begin{cases} y_{K-1,i} & \text{if } j = l, \\ 0 & \text{otherwise.} \end{cases} \end{aligned}$$

For sigmoid output units, the factors in the partial derivative $\frac{\partial J}{\partial z_{Kl}}$ can be computed as

$$\frac{\partial J_{\text{BCE}}}{\partial y_{Kc}} = \begin{cases} -\dfrac{1}{C}\dfrac{1}{y_{Kc}} & \text{if } c = c^*, \\ -\dfrac{1}{C}\dfrac{-1}{(1 - y_{Kc})} & \text{otherwise,} \end{cases}$$

$$\frac{\partial y_{Kc}}{\partial z_{Kl}} = \begin{cases} y_{Kc}\,(1 - y_{Kc}) & \text{if } l = c, \\ 0 & \text{otherwise,} \end{cases}$$

while for softmax output units, these factors can be computed as

$$\frac{\partial J_{\text{CCE}}}{\partial y_{Kc}} = \begin{cases} -\dfrac{1}{C}\dfrac{1}{y_{Kc}} & \text{if } c = c^*, \\ 0 & \text{otherwise,} \end{cases}$$

$$\frac{\partial y_{Kc}}{\partial z_{Kl}} = \begin{cases} y_{Kc}\,(1 - y_{Kc}) & \text{if } l = c, \\ -\,y_{Kc}\,y_{Kl} & \text{otherwise.} \end{cases}$$

In both cases, it results that

$$\frac{\partial J}{\partial z_{Kl}} = \begin{cases} -\dfrac{1}{C}\,(1 - y_{Kl}) & \text{if } l = c^*, \\ -\dfrac{1}{C}\,(-y_{Kl}) & \text{otherwise,} \end{cases}$$

which is equivalent to

$$\frac{\partial J}{\partial z_K} = -\frac{1}{C}\,(y^* - y_K) = -\frac{e}{C},$$

where e is the error vector. Therefore, the weight updates in the output layer can be rewritten as

$$W_K \leftarrow W_K + \frac{\eta}{C} e y_{K-1}^T.$$

In the hidden layers, the weight updates follow

$$W_k \leftarrow W_k - \eta \delta y_k y_{k-1}^T.$$

On the one hand, if the training relies on the BP algorithm, the modulatory signals δz_k, which are equivalent to the estimated loss gradients δy_k in the linear case, correspond to the loss function gradient:

$$\delta y_k = \delta z_k = \frac{\partial J}{\partial y_k} = -\frac{1}{C}\left(\prod_{i=k+1}^{K} W_i^T\right) e.$$

On the other hand, if the DRTP algorithm is used, the modulatory signals are projections of the one-hot-encoded target vector y^* through fixed random connectivity matrices B_k:

$$\delta y_k = \delta z_k = B_k^T y^*.$$

In order to provide learning, the modulatory signals prescribed by BP and DRTP must be within 90° of each other, i.e. their dot product must be positive:

$$-e^T \left(\prod_{i=k+1}^{K} W_i^T \right)^T B_k^T y^* > 0.$$

Lemma. In the case of zero-initialized weights, i.e. $W_k^0 = 0$ for $k \in [1, K]$, $k \in \mathbb{N}$, and hence of zero-initialized hidden layer outputs, i.e. $y_k^0 = 0$ for $k \in [1, K-1]$ and $z_K^0 = 0$, considering a DRTP-based training performed recursively with a single element of the training set (x, c^*) and y^* denoting the one-hot encoding of c^*, at every discrete update step t, there are non-negative scalars $s_{y_k}^t$ and $s_{W_k}^t$ for $k \in [1, K-1]$ and a C-dimensional vector $s_{W_K}^t$ such that

$$\begin{aligned}
y_k^t &= -s_{y_k}^t \left(B_k^T y^* \right) && \text{for} \quad k \in [1, K-1] \\
W_1^t &= -s_{W_1}^t \left(B_1^T y^* \right) x^T \\
W_k^t &= s_{W_k}^t \left(B_k^T y^* \right) \left(B_{k-1}^T y^* \right)^T && \text{for} \quad k \in [2, K-1] \\
W_K^t &= -s_{W_K}^t \left(B_{K-1}^T y^* \right)^T .
\end{aligned}$$

Proof. The lemma is proven by induction.

For $t = 0$, the conditions required to satisfy the lemma are trivially met by choosing $s_{y_k}^0, s_{W_k}^0 = 0$ for $k \in [1, K-1]$, and $s_{W_K}^0$ as a zero vector, given that $y_k^0 = 0$ for $k \in [1, K-1]$ and $W_k^0 = 0$ for $k \in [1, K]$.

For $t > 0$, considering that the conditions are satisfied at a given discrete update step t, it must be shown that they still hold at the next discrete update step $t+1$. In the hidden layers, the weights are updated using the modulatory signals prescribed by DRTP. For the first hidden layer, we have

$$\begin{aligned}
W_1^{t+1} &= W_1^t - \eta B_1^T y^* x^T \\
&= -s_{W_1}^t \left(B_1^T y^* \right) x^T - \eta \left(B_1^T y^* \right) x^T \\
s_{W_1}^{t+1} &= s_{W_1}^t + \eta \;= s_{W_1}^t + \Delta s_{W_1}^t
\end{aligned}$$

and for subsequent hidden layers, i.e. for $k \in [2, K-1]$, we have

$$\begin{aligned} W_k^{t+1} &= W_k^t - \eta B_k^T y^* y_{k-1}^{tT} \\ &= s_{W_k}^t \left(B_k^T y^*\right) \left(B_{k-1}^T y^*\right)^T + \eta s_{y_{k-1}}^t \left(B_k^T y^*\right) \left(B_{k-1}^T y^*\right)^T \\ s_{W_k}^{t+1} &= s_{W_k}^t + \eta s_{y_{k-1}}^t \ = s_{W_k}^t + \Delta s_{W_k}^t. \end{aligned}$$

The weights in the output layer are updated according to the loss function gradient, thus leading to

$$\begin{aligned} W_K^{t+1} &= W_K^t + \frac{\eta}{C} \left(y^* - y_K^t\right) y_{K-1}^{tT} \\ &= W_K^t - \frac{\eta}{C} \left(y^* - y_K^t\right) s_{y_{K-1}}^t \left(B_{K-1}^T y^*\right)^T \\ &= -s_{W_K}^t \left(B_{K-1}^T y^*\right)^T - \frac{\eta s_{y_{K-1}}^t}{C} \left(y^* - y_K^t\right) \left(B_{K-1}^T y^*\right)^T \\ s_{W_K}^{t+1} &= s_{W_K}^t + \frac{\eta s_{y_{K-1}}^t}{C} \left(y^* - y_K^t\right). \end{aligned}$$

The output of the first hidden layer is

$$\begin{aligned} y_1^{t+1} &= W_1^{t+1} x \\ &= \left(W_1^t - \eta B_1^T y^* x^T\right) x \\ &= \underbrace{W_1^t x}_{y_1^t} - \eta x^T x \left(B_1^T y^*\right) \\ &= -s_{y_1}^t \left(B_1^T y^*\right) - \eta \left\|x\right\|^2 \left(B_1^T y^*\right) \\ s_{y_1}^{t+1} &= s_{y_1}^t + \eta \left\|x\right\|^2 \ = s_{y_1}^t + \Delta s_{y_1}^t \end{aligned}$$

and the output of the k-th hidden layer for $k \in [2, K-1]$ is given by

$$\begin{aligned} y_k^{t+1} &= W_k^{t+1} y_{k-1}^{t+1} \\ &= -s_{W_k}^{t+1} \left(B_k^T y^*\right) \left(B_{k-1}^T y^*\right)^T s_{y_{k-1}}^{t+1} \left(B_{k-1}^T y^*\right) \\ &= -\left(s_{W_k}^t + \eta s_{y_{k-1}}^t\right) \left(s_{y_{k-1}}^t + \Delta s_{y_{k-1}}^t\right) \left\|B_{k-1}^T y^*\right\|^2 \left(B_k^T y^*\right) \\ &= -\underbrace{s_{W_k}^t s_{y_{k-1}}^t \left\|B_{k-1}^T y^*\right\|^2}_{s_{y_k}^t} \left(B_k^T y^*\right) - \left(s_{W_k}^t \Delta s_{y_{k-1}}^t + \eta s_{y_{k-1}}^t \left(s_{y_{k-1}}^t + \Delta s_{y_{k-1}}^t\right)\right) \left\|B_{k-1}^T y^*\right\|^2 \left(B_k^T y^*\right) \\ s_{y_k}^{t+1} &= s_{y_k}^t + \left(s_{W_k}^t \Delta s_{y_{k-1}}^t + \eta s_{y_{k-1}}^t \left(s_{y_{k-1}}^t + \Delta s_{y_{k-1}}^t\right)\right) \left\|B_{k-1}^T y^*\right\|^2 \ = s_{y_k}^t + \Delta s_{y_k}^t. \end{aligned}$$

The coefficients $s^t_{W_1}$ and $s^t_{y_1}$ are updated with strictly positive quantities $\Delta s^t_{W_1}$ and $\Delta s^t_{y_1}$ at each update step t and are thus strictly positive for $t > 0$. Furthermore, the coefficients $s^t_{W_k}$ and $s^t_{y_k}$ are updated based on the coefficients of the previous layer and will therefore be strictly positive for $k \in [1, K-1]$. □

Theorem. Under the same conditions as in the lemma and for the linear-hidden-layer network dynamics described above, the k-th layer modulatory signals prescribed by DRTP are always a negative scalar multiple of the Moore-Penrose pseudo-inverse of the product of forward matrices of layers $k+1$ to K, located in the feedback pathway between the output layer and the k-th hidden layer, multiplied by the error. That is, for $k \in [1, K-1]$ and $t > 0$,

$$-\frac{1}{s^t_k}\left(\prod_{i=K}^{k+1} W^t_i\right)^+ e = B^T_k y^* \quad \text{with} \quad s^t_k > 0.$$

Proof. When replacing the forward weights W^t_i by the expressions given in the lemma, the above equality becomes

$$\left[\left(\prod_{i=K-1}^{k+1} s^t_{W_i}\right) s^t_{W_K}\left(\prod_{i=K-1}^{k+1}\left\|B^T_i y^*\right\|^2\right)\left(B^T_k y^*\right)^T\right]^+ \left(y^* - y^t_K\right) = s^t_k B^T_k y^*$$

$$\left(\prod_{i=k+1}^{K-1} s^t_{W_i}\right)^{-1}\left(\prod_{i=k+1}^{K-1}\left\|B^T_i y^*\right\|^2\right)^{-1}\left[s^t_{W_K}\left(B^T_k y^*\right)^T\right]^+ \left(y^* - y^t_K\right) = s^t_k B^T_k y^*$$

$$\left(\prod_{i=k+1}^{K-1} s^t_{W_i}\right)^{-1}\left(\prod_{i=k+1}^{K-1}\left\|B^T_i y^*\right\|^2\right)^{-1}\left(B^T_k y^*\right)^{T+} s^{t+}_{W_K}\left(y^* - y^t_K\right) = s^t_k B^T_k y^*$$

$$\left(\prod_{i=k+1}^{K-1} s^t_{W_i}\right)^{-1}\left(\prod_{i=k+1}^{K-1}\left\|B^T_i y^*\right\|^2\right)^{-1}\underbrace{\left\|B^T_k y^*\right\|^{-2}\left(B^T_k y^*\right)}_{\left(B^T_k y^*\right)^{T+}}\underbrace{\left\|s^t_{W_K}\right\|^{-2} s^{tT}_{W_K}}_{s^{t+}_{W_K}}\left(y^* - y^t_K\right) = s^t_k B^T_k y^*$$

$$\left(\prod_{i=k+1}^{K-1} s^t_{W_i}\right)^{-1}\left(\prod_{i=k}^{K-1}\left\|B^T_i y^*\right\|^2\right)^{-1}\left\|s^t_{W_K}\right\|^{-2} s^{tT}_{W_K}\left(y^* - y^t_K\right)\left(B^T_k y^*\right) = s^t_k\left(B^T_k y^*\right).$$

By identification, it is found that

$$s^t_k = \frac{s^{tT}_{W_K}\left(y^* - y^t_K\right)}{\left(\prod_{i=k+1}^{K-1} s^t_{W_i}\right)\left(\prod_{i=k}^{K-1}\left\|B^T_i y^*\right\|^2\right)\left\|s^t_{W_K}\right\|^2}.$$

From the lemma proof, the update formula for the vector $s^t_{W_K}$ is given by

$$s^{t+1}_{W_K} = s^t_{W_K} + \frac{\eta s^t_{y_{K-1}}}{C}\left(y^* - y^t_K\right),$$

where η, C and $s^t_{y_{K-1}}$ are positive scalars. In the framework of classification problems where outputs are strictly bounded between 0 and 1, for any example (x, c^*) in the training set, the error vector $e = \left(y^* - y^t_K\right)$ has a single strictly positive entry $(1 - y_{Kc})$ at the class label index $c = c^*$, all the other entries $-y_{Kc}$ with $c \neq c^*$ being strictly negative. This sign information is constant as the network is trained with a single training example. Given that $s^0_{W_K} = 0$ from zero-weight initialization and that $s^t_{W_K}$ is updated in the same direction as e, we have at every discrete update step t

$$\text{sign}\left(s^t_{W_K}\right) = \text{sign}\left(y^* - y^t_K\right),$$

and thus

$$s^{tT}_{W_K}\left(y^* - y^t_K\right) > 0.$$

Therefore, the scalars s^t_k are strictly positive for $t > 0$. □

Alignment. In the framework of classification problems, as the coefficients s^t_k are strictly positive scalars for $t > 0$, it results from the theorem that the dot product between the BP and DRTP modulatory signals is strictly positive, i.e.

$$-e^T \left(\prod_{i=k+1}^{K} W_i^T\right)^T \left(B_k^T y^*\right) > 0$$

$$e^T \underbrace{\left(\prod_{i=k+1}^{K} W_i^T\right)^T \left(\prod_{i=K}^{k+1} W_i\right)^+}_{I} \frac{e}{s^t_k} > 0$$

$$\frac{e^T e}{s^t_k} > 0.$$

The BP and DRTP modulatory signals are thus within 90° of each other. □

REFERENCES

Lillicrap, T. P., Cownden, D., Tweed, D. B., and Akerman, C. J. (2016). Random synaptic feedback weights support error backpropagation for deep learning. *Nature Communications* 7, 1–10. doi:10.1038/ncomms13276
